# Metagenomics of the MAST-3 stramenopile, Incisomonas, and its associated microbiome reveals unexpected metabolic attributes and extensive nutrient dependencies

**DOI:** 10.1099/mgen.0.001510

**Published:** 2025-11-13

**Authors:** Dominic E. Absolon, Victoria L. N. Jackson, Adam Monier, Alison G. Smith, Katherine E. Helliwell

**Affiliations:** 1 Department of Plant Sciences, University of Cambridge, Cambridge CB2 3EA, UK; 2 Biosciences, Faculty of Health and Life Sciences, University of Exeter, Exeter, EX4 4QD, UK; 3 Living Systems Institute, University of Exeter, Exeter, EX4 4QD, UK; 4 Marine Biological Association, Citadel Hill, Plymouth, PL1 2PB, UK

**Keywords:** DMSP, *Incisomonas*, MASTs, protists, stramenopiles, vitamins

## Abstract

Protists are polyphyletic single-celled eukaryotes that underpin global ecosystem functioning, particularly in the oceans. Most remain uncultured, limiting the investigation of their physiology and cell biology. MArine STramenopiles (MASTs) are heterotrophic protists that, although related to well-characterized photosynthetic diatoms and parasitic oomycetes, are poorly studied. The Nanomonadea (MAST-3) species *Incisomonas marina* has been maintained in co-culture with a bacterial consortium, offering opportunities to investigate the metabolic attributes and nutritional dependencies of the community. Employing a metagenomics approach, the 68 Mbp haploid genome of *I. marina* was retrieved to an estimated completeness of 93%, representing the most complete MAST genome so far. We also characterized the diversity of, and assembled genomes for, 23 co-cultured bacteria. Auxotrophy of *I. marina* for B vitamins (B_1_, B_2_, B_6_, B_7_ and B_12_), but not vitamins C, B_3_, B_5_ and B_9_, was predicted. Several bacteria also lacked complete B-vitamin biosynthesis pathways, suggesting that vitamins and/or their precursors are exchanged in the consortium. Moreover, *I. marina* lacked the ability to synthesize half the protein amino acids, although genes encoding the complete urea cycle were identified, like diatoms; this may play a role in recycling organic nitrogen compounds. Unexpectedly, we also identified the gene *DSYB* for dimethylsulphoniopropionate biosynthesis. Biosynthesis of this important stress protectant and bacterial chemoattractant is typically found in photosynthetic eukaryotes and has not been identified before in heterotrophic stramenopiles. Together, our study reveals the metabolic attributes of a hitherto understudied organism, advancing knowledge of the evolution and adaptations of the stramenopiles and informing future culturing efforts.

**Impact Statement**

Protists play a crucial role in ocean ecosystems, yet limited genomic resources and culturing challenges have hindered basic understanding of their biology and genomic attributes. Here, we report the most complete genome to date for a member of the abundant group of marine protists – the MArine STramenopiles (MASTs). We employed metagenomics analysis of the Nanomonadea (MAST-3) *Incisomonas marina* and its associated bacterial consortium. We identified widespread auxotrophy of *I. marina* for B vitamins and amino acids, which must be obtained from co-habiting bacteria or other environmental sources. However, contrary to prevailing views on the nutritional needs of heterotrophic protists, *I. marina* can synthesize multiple vitamins itself. Unexpectedly, *I. marina* also encodes genes for dimethylsulphoniopropionate (DMSP) biosynthesis. This provides the first evidence of a role of heterotrophic stramenopiles in global DMSP cycling and brings new insights on the evolution of DMSP biosynthesis in eukaryotes. Together, our study helps establish *I. marina* as a model heterotrophic stramenopile with robust sequence resources and supports future culturing efforts of MASTs.

## Data Availability

All metagenomics data for this study have been deposited to the European Nucleotide Archive using the accession number ERZ27257279 for the entire metagenome. The accession numbers for the individual bins are provided in Table S9.

## Introduction

Marine protists play crucial roles in nutrient and carbon cycling in the oceans [1]. However, the majority of heterotrophic eukaryotic micro-organisms remain uncharacterized, their existence evident only through molecular surveys using DNA metabarcoding approaches [2]. A major polyphyletic assemblage described within this unexplored protistan diversity is the MArine STramenopiles, so-called ‘MASTs’ [3]. An emerging picture from environmental surveys (e.g. *Tara* Oceans [4]) is that MASTs are abundant and globally distributed across marine habitats [5,8]. MASTs occupy several independent clades at the base of the stramenopile lineage, which encompasses a diverse range of eukaryotes. These include well-studied groups such as the heterotrophic oomycetes, a clade containing many parasitic species [9], and thraustochytrids, of biotechnological importance as a source of omega-3 fatty acids [10]. Moreover, there are many globally significant photosynthetic members, including diatoms and brown macroalgae; the diatoms alone are estimated to be responsible for ~20% of planetary carbon fixation [11].

In contrast, the different lifestyles and evolutionary origins of MAST species are poorly understood. Twenty-one distinct MAST clades have been described based on 18S rRNA gene analysis [6,12], and they reportedly exhibit a range of different ecological strategies, including symbioses with diatoms and bacterial grazing (bacteriovory) [9,13]. Knowledge of the fundamental biology of MASTs has been hindered considerably by cultivation difficulties, likely due to their nutritional requirements, including reliance on other microbes in their environment [2]. Just two MAST species have been cultured to date: *Incisomonas marina* (from the MAST-3 clade) [14] and *Pseudophyllomitus vesiculosus* (MAST-6) [15], both with associated microbiota. Currently, *I. marina* is the only publicly available strain. Moreover, genome sequencing efforts have generally relied upon single-cell assembled genomes (SAGs) and metagenome-assembled genomes (MAGs), recovered from environmental sampling approaches [4,16]. A major challenge of such techniques is poor genome recovery, with genome completeness ranging from 83% to just 7% [16,17]. These studies have yielded details of some of the functional repertoire of MASTs, including aspects relating to motility, the phagocytosis apparatus, carbohydrate-active (CaZY) enzymes and rhodopsin-encoding genes [4,16, 17], and phylogenomic analysis of *I. marina* has been important in resolving aspects of stramenopile phylogeny [18]. However, whilst these studies have examined the presence of particular gene families, assessing key metabolic capabilities, such as energy utilization and storage pathways, or biosynthesis of amino acids and cofactors that would indicate their nutritional needs and ecological lifestyle, is more challenging.

The diversity and wide array of ecological lifestyles represented by the stramenopiles make this group ideal to study evolutionary transitions, including from heterotrophy and/or mixotrophy to autotrophy [19], and indeed back again [20]. Photosynthesis arose within the stramenopiles through endosymbiotic acquisition of a red algal-derived plastid, driving genetic mixing between the protistan host and algal endosymbiont [21,22]. The combination of metabolic attributes likely contributed to the ecological success of the diatoms, as in other major marine algal taxa such as dinoflagellates and haptophytes [23]. We now know that despite being photoautotrophs, diatoms take up significant amounts of organic substrates [24], although whether this capacity has been shaped by their heterotrophic ancestry requires further exploration. Just as organic substrate usage may not be accurately predicted by inferred nutritional mode, the capacity for biosynthesis of specific metabolites need not be either. Many obligate heterotrophs (e.g. fungi and bacteria) often synthesize vitamins [25,26], yet the majority of algae are known to be auxotrophic for at least one B vitamin despite being photoautotrophic [27]. These compounds have emerged as important metabolites mediating microbial eukaryote–bacteria interactions [28,30]. B vitamins have even been predicted to flow from a bacterivorous protist (choanoflagellate) to bacterial associates, challenging typical models of trophic interactions in marine food webs [31]. Similarly, amino acids and signalling molecules like dimethylsulphoniopropionate (DMSP) [32,33], made by many photosynthetic stramenopiles [34,35], are widely exchanged. However, the biosynthetic capabilities of most MASTs are unknown, limiting understanding of their role in the broader marine ecosystems. Establishing the nutritional needs of MASTs is also crucial to facilitate culturing efforts.

Here, we took advantage of the publicly available *I. marina* co-culture and characterized it at the physiological, morphological and genome sequence level, allowing detailed examination of the specific nutritional dependencies of *I. marina* and the co-cultured bacterial consortium, together with the identification of novel gene functions. This work helps establish *I. marina* as a model heterotrophic stramenopile with robust sequencing resources.

## Methods

### Culturing of *I. marina*

*I. marina* was acquired from the Culture Collection of Algae and Protozoa (culture number CCAP 997/1). The culture was maintained in Artificial Seawater for Protozoa (ASWP; https://www.ccap.ac.uk/index.php/media-recipes/), made up with a single barley grain, sterilized by boiling (10 min), and autoclaved soil extract (SE2). These additions had previously been shown to be required as a source of carbon and undefined micronutrients [14] for culture viability. Cultures were routinely maintained at 15 °C in a 12 : 12 light:dark cycle, sub-culturing every 16 weeks (as recommended by CCAP). For experimental work, cultures were transferred more frequently (every 2 weeks) to ensure that cells were actively growing.

### Bacterial isolation and colony PCR

For the initial analysis of bacteria present in *I. marina* cultures, samples were spread on marine broth (Difco) agar plates and incubated in the conditions described above. Colony PCR was performed on colonies with distinct morphological appearances to amplify 16S rRNA gene (V6-V8) sequences, using ACGCGHNRAACCTTACC (forward) and ACGGGCRGTGWGTRCAA (reverse) primers. PCR was carried out using Red Taq kit (Sigma-Aldrich), and the cycling conditions were 95 °C for 1 min followed by 30 cycles of 95 °C for 0.5 min, 50 °C for 0.5 min and 72 °C for 1.5 min, followed by a final extension at 70 °C for 3 min. PCR products were analysed using a 2% agarose gel. Illustra GFX Gel Band Purification kit was used to purify PCR products that served as templates for Sanger sequencing (Source BioScience, Cambridge, UK). Resulting sequences were analysed in Geneious Prime (2022.0.2) and the silva incremental aligner (1.7.2) taxonomy identification tool [36].

### Antibiotic treatment of the *I. marina* consortium

To investigate the dependency of *I. marina* on the bacterial consortium, cultures were treated with two different antibiotic cocktails for 7 days (Table S1, available in the online Supplementary Material). The presence of bacteria was assessed by streaking out cultures onto marine broth (Difco) agar plates. Additionally, cultures were visualized via light microscopy, comparing treated and non-treated cells, as well as a medium-only control, i.e. that was not inoculated with the *I. marina* consortium.

### Scanning electron microscopy

Cells were grown on Melinex plastic coverslips (Agar Scientific) for 14 days, then briefly dipped twice in cold, de-ionized water to remove any buffer salts and quickly plunge-frozen in liquid nitrogen-cooled ethane. Samples were transferred to liquid nitrogen-cooled brass inserts and freeze-dried overnight in a liquid nitrogen-cooled turbo freeze-drier (Quorum K775X). Samples were mounted on aluminium scanning electron microscopy (SEM) stubs using conductive silver paint (Agar Scientific) and coated with 15 nm iridium using a Quorum K575X sputter coater. Samples were viewed using an FEI Verios 460 scanning electron microscope run at 2.00 keV and 50 pA probe current. Secondary electron images were acquired using either an Everhard–Thornley detector in field-free mode (low resolution) or a through-lens detector in full immersion mode (high resolution).

### Metagenomics of *I. marina* culture

#### DNA extraction

*I. marina* cells (grown in six 200 ml cultures) were concentrated via centrifugation, resulting in a pellet consisting of a top brown layer and a lower white layer. Microscopy confirmed *I. marina* cells to be in the brown fraction. To reduce the bacterial load, which might interfere with sequence acquisition, these two fractions were manually separated, and DNA was extracted from the brown fraction using a phenol-chloroform extraction. Biomass was resuspended in 400 µl of nuclease-free water, and 400 µl of SDS elution buffer (2% SDS, 100 mM Tris-HCl pH 8.0, 400 mM NaCl, and 40 mM EDTA, pH 8) was added. The resulting sample was vortexed, before 800 µl of phenol:chloroform with iso-amyl alcohol (25 : 24 : 1 v/v) was added. The liquid suspension was vortexed and then centrifuged for 5 min at 14,000 ***g***, and the top phase was transferred to a 2 ml Eppendorf tube. This was repeated twice before the addition of 800 µl of chloroform:iso-amyl (24 : 1 v/v). The sample was then vortexed for 2 min, before being centrifuged for 5 min at 14,000 ***g***, and the top phase was transferred to a new 2 ml Eppendorf tube. To this, 2.5× volume (~1.4 ml) of 100% ethanol (−20 °C) was added, and the sample was incubated overnight at −20 °C. The sample was centrifuged for 30 min at 16,000 ***g*** at 4 °C, and the supernatant discarded. The pellet was resuspended in 400 µl of TE buffer, 1 µl of RNase A added, and incubated for 1 h at 37 °C before one further phenol-chloroform extraction and ethanol precipitation. DNA was quality-checked using a Nanodrop (Thermo Fisher) and Qubit dsDNA HS assay kit (Thermo Fisher).

### Long-read sequencing, quality control and genome assembly

The sequencing library was prepared using the Oxford Nanopore Technologies (ONT) ligation sequencing kit (SQK-LSK109) and the NEBNext FFPE DNA repair mix (M6630; New England Biolabs), NEBNext Ultra II end repair/dA-tailing module (E7546; NEB) and NEBNext quick ligation module (E6056; NEB), following the ONT protocol (version GDE_9063_V109_REVV_14AUG2019) with a modified incubation time for the repair and end-prep reaction. Briefly, DNA repair and end-prep reagents were added to 1 µg DNA and incubated for 20 min at 20 °C, followed by a final incubation step of 5 min at 65 °C. Adapter ligation and clean-up were carried out according to the ONT protocol using long fragment buffer, and the eluted library was incubated at 37 °C for 10 min to improve the recovery of long fragments. Approximately 800 ng of DNA library was loaded onto a MinION SpotON flow cell (R9.4.1), and sequencing was carried out over 72 h. Base calling was performed with Guppy (ONT) (4.2.3) in high-accuracy mode (HAC model dna_r9.4.1_450bps_hac). The quality of the long-read data was assessed with LongQC (1.2.0) [37] and nanoplot (1.34.0) [38], both run with default parameters. Trimmomatic (0.39) [39] was used for read trimming, and porechop (0.2.4) (https://github.com/rrwick/Porechop) was used to remove adapter sequences [40], both with default parameters.

The long-read data was assembled with Flye (2.8.3) [41], run with the read input flag ‘--nano-raw’ and the flag ‘--meta’. The resulting *de novo* assembly was polished using Illumina short-read data from [18] using Pilon (1.24) [42]. Polishing was performed iteratively three times. The resulting assembly was run through the NCBI Foreign Contamination Screen [43] prior to running downstream analysis. The assembled contigs were also run through Tiara (1.0.3) [44] to identify organellar sequences, but none were identified. Ploidy level was assessed using Genomescope2 (2.0) [45].

### Anvi’o metagenomics workflow and metabolic pathway analysis

The general Anvi’o (v7) workflow can be found at https://merenlab.org/2016/06/22/anvio-tutorial-v2/. Briefly, the long reads used for the initial assembly were aligned back to the final assembly using LongReadAligner (lra, 1.1.2) [46]. This allowed the calculation of read recruitment for future bins and for Anvi’o to perform hierarchical clustering. The resulting SAM file was then converted to a BAM file and a BAM index file using samtools (1.12) [47]. From there, Anvi’o [48] was used to create a contigs database (anvi-gen-contigs-database), followed by searching for Hidden Markov Models (HMMs) of rRNA genes and single-copy genes for protists and bacterial lineages (anvi-run-hmms). An Anvi’o profile (anvi-profile) was then created for the contigs database to allow the use of the Anvi’o interactive interface for supervised binning (anvi-interactive [48]). Binning was performed manually based on read clustering, presence of rRNA genes, differences in GC content and read coverage. MAGs were extracted by summarizing the binning effort (anvi-summarize).

Further analysis of the bacterial MAGs was performed with the Anvi’o suite of analysis programs. A contigs database was created for each MAG (anvi-gen-contigs-database) before repeating the HMM search for rRNA genes and single-copy genes (anvi-run-hmms). The level of completeness was assessed for each bin by running anvi-estimate-genome-completeness, which measures the number of single-copy orthologues identified compared to the full set. Taxonomic assignment for each bin was performed with anvi-estimate-scg-taxonomy, which uses single-copy core gene hits to the Genome Taxonomy Database (GTDB) [49] to assign taxonomy (with the exception of the eukaryotic bin known to be *I. marina* as confirmed by blast hits to the 18S sequence on the NCBI).

Although Anvi’o performs *ab initio* gene calling on binned contigs, using the program prodigal [50], this is optimized for prokaryotic gene calling. Instead, to achieve an appropriate set of gene calls for the eukaryotic MAG, contigs were submitted to the Augustus web server (https://bioinf.uni-greifswald.de/webaugustus/). Subsequent assessment of genome completeness was performed using BUSCO (v5 – stramenopile_odb10). Prediction of subcellular localization of proteins was conducted using HECTAR [51].

### Searching Tara Oceans eukaryotic metagenomes and single-cell assembled genomes

The DSYB protein sequence from *I. marina* (Dataset S1) was searched against the EUK_SMAGs database (ie both SAGs and MAGs) via the *Tara Ocean* Gene Atlas portal [52], using a stringent e-value cut-off of 1e^−70^, with abundance plotted as the percent of total reads. DSYB hits were further scrutinized by constructing maximum likelihood trees together with the *I. marina* query sequence, as well as DSYB sequences of eukaryotic phytoplankton and bacterial taxa from [34,53], including functionally validated sequences. Geographic distributions were subsequently plotted for surface waters (SRF) and the deep chlorophyll maxima (DCM).

### Maximum likelihood tree construction of DSYB sequences

Multiple sequence alignments of DSYB/DsyB protein sequences [34,53] were generated using MAFFT (7.520) [54]. Alignments were trimmed in auto mode by trimal (1.4.1) [55], where sites made up of 60% gaps were removed. A maximum likelihood tree was then produced with IQTREE [56] with the following parameters: iqtree -s infile.txt -bb 10000 -safe -bnni -alrt 10000 -st AA - seed 1000 -msub nuclear -t RANDOM -nt AUTO -pre out -m TEST (Q.pfam+I+R5 was determined to be the best fit model). The raw FASTA file of all sequences used to construct the phylogenetic tree is given in Dataset S1. The final trimmed alignment used for tree construction was 365 amino acids in length.

### Genome annotations

Genome annotation was used to compare the functional landscape of *I. marina* against other more widely studied stramenopiles. The genomes of *Phaeodactylum tricornutum*, *Schizochytrium aggregatum* ATCC 28209, *Phytophthora sojae*, *Cafeteria roenbergensis* BVI and *Blastocystis hominis* (Singapore isolate B) were acquired from the JGI genome browser. The eggNOG-mapper v2 annotation server (http://eggnog-mapper.embl.de/) [57] was used to run gene annotation on each genome using default settings. Subsequent KEGG Orthology annotation data were plotted using the venny4py Python package (https://github.com/timyerg/venny4py).

Estimation of the metabolic capacity of organisms in the community, both eukaryotic and prokaryotic, was achieved using the KEGG orthologue (KO) annotation from ghostKOALA [58] and the KEGG Mapper-Reconstruct tool (https://www.genome.jp/kegg/mapper/reconstruct.html) to overlay KOs on biosynthetic pathways, followed by manual inspection to determine whether they were likely complete. Taxonomic assignment of the closest related KO for each annotated protein was also extracted from ghostKOALA output and used to identify protein sequences with close similarity to red algal protein sequences.

## Results and discussion

### Imaging and physiology of the *I. marina*–bacterial consortium

**Fig. 1.**
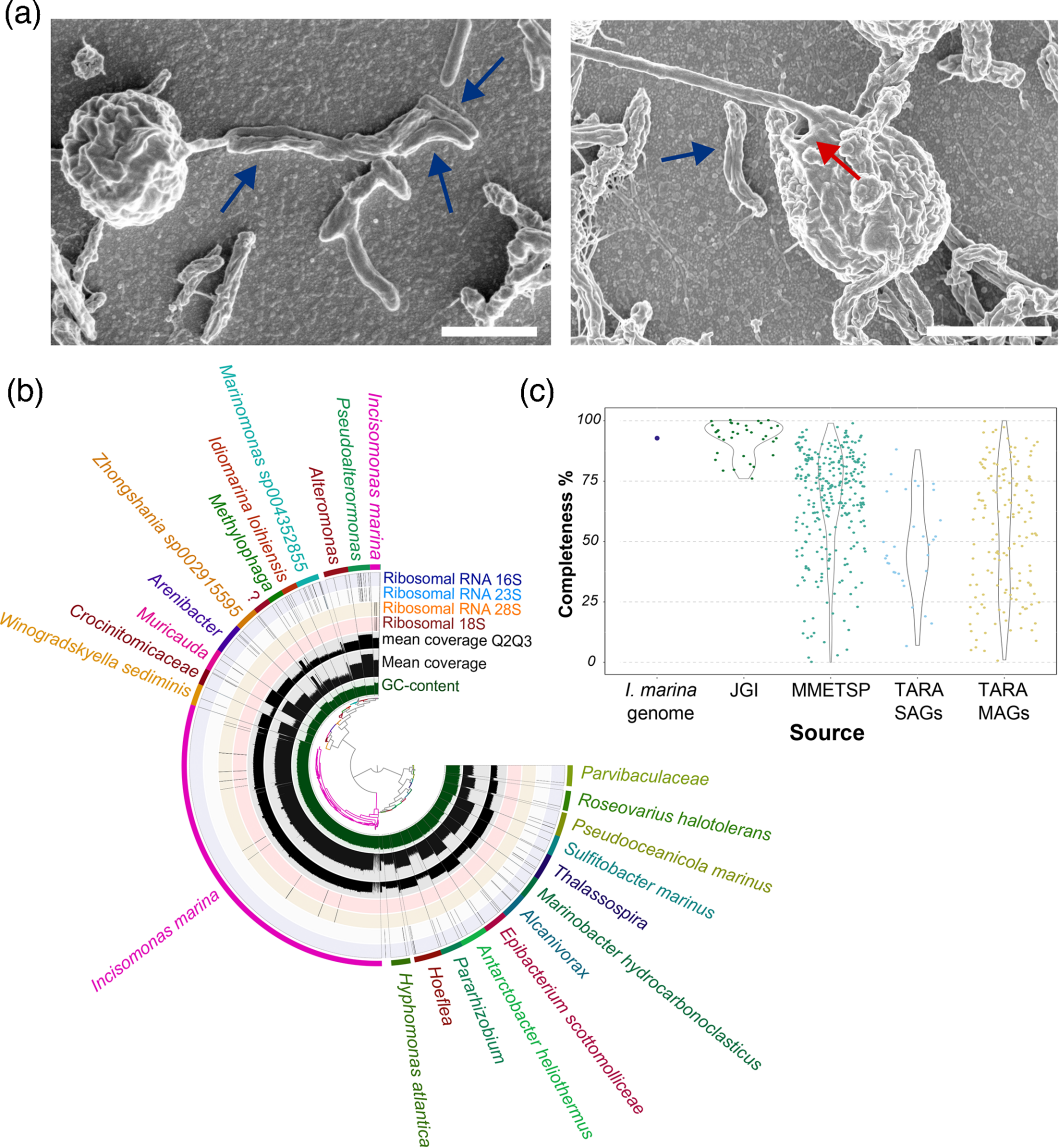
Imaging and metagenomic analysis of *I. marina* and its associated bacterial community. (a) SEM of *I. marina*. Individual *I. marina* cells displaying a single flagellum, as well as physically close associations with bacteria, including along the length of the flagellum; blue arrows indicate bacterial cells. The red arrow indicates a consistently observed opening at the base of the flagellum, as well as an ‘overhanging lip’ as observed by [14]. Scale bar: 2 µm. (b) A view of the Anvi'o interactive interface used to perform supervised binning for MAG recovery. The central tree is a hierarchical clustering of re-aligned long reads to the final metagenome assembly. Each track surrounding this tree represents (in order from inside out) GC content, mean coverage (read recruitment), mean coverage for Q2 and Q3 and locations of rRNA genes for 18S, 28S, 23S and 16S. Each bin is coloured individually by the final outermost track with an associated taxonomic prediction where possible. (c) Comparison of the genome completeness estimations for available stramenopile genomes and transcriptomes (the latter from the Marine Microbial Eukaryote Transcriptome Sequencing Project, MMETSP [76]) versus the estimated completeness of the *I. marina* MAG generated in this study (93%), measured by BUSCO (**v5**) [77] against stramenopile_odb10.

The Nanomonadea species *I. marina* (CCAP 977/1) was originally isolated from an estuarine environment with a consortium of bacteria [14], with which it has been maintained ever since. Due to the lack of cultured MASTs, microscopy studies of these organisms are limited. We therefore first inspected *I. marina* with its associated bacterial assemblage using bright-field microscopy. A mixed population of protistan and bacterial cells was clearly visible (Fig. S1A). SEM revealed physical associations between *I. marina* and bacteria, including bacterial attachment along the length of the protistan flagellum (Fig. 1a, left image). We also observed clumping and biofilm formation, particularly in older cultures (Fig. S1B–D), as well as evidence of dead and decaying *I. marina* cells, with associated bacteria in close proximity, potentially feeding on the *I. marina* biomass (red arrow, Fig. S1E). Our images corroborate earlier descriptions of *I. marina* as a nanoflagellate of 2–3 µm with a single smooth flagellum [14] (Figs 1a and S1F). Additionally, we identified the presence of an opening at the flagellum base where it joins the cell body (red arrow) (Fig. 1a, right image). Initial analysis of the bacterial community by plating on marine broth agar identified four morphologically different colonies. Analysis of the 16S rRNA genes from samples of these colonies identified them as *Ruegeria* sp., *Gammaproteobacteria bacterium*, *Winogradskyella* sp., *Marinobacter hydrocarbonoclasticus* and *Pseudooceanicola marinus*.

To investigate the dependency of *I. marina* on the bacteria present versus the organic material provided from the culture medium (ASWP medium that includes soil extract as well as a boiled barley grain, detailed in Methods), cultures grown in ASWP were treated with two different antibiotic cocktails (Table S1). The cultures were imaged and compared to a control with no antibiotics, as well as another media-only control (no cells), over a time course of 7 days. After 5 days of antibiotic treatment, no *I. marina* cells were visible (Fig. S2A, B). Likewise, no bacterial growth was observed on marine broth agar plates inoculated with antibiotic-treated cultures, compared to the untreated control (Fig. S2C). Whilst it is possible that the antibiotics killed *I. marina* directly, the antibiotics chosen for this study have been used widely to minimize bacterial contamination of other stramenopile cultures [59,63], and we used lower concentrations than those reported in the literature (Table S1). Thus, these observations suggest that removal of the bacterial community from the culture by antibiotics resulted in loss of a nutritional source from *I. marina*, leading to the death of the protist.

### Metagenomics analysis of *I. marina* and its associated bacterial community

To investigate further *I. marina* and its microbiota, we carried out metagenomic sequencing of the entire consortium, using Oxford Nanopore long-read sequencing. This yielded 1,415,560 reads spanning a total of 16,635,245,180 bases with a read N50 of 18,816 bases. A supervised binning effort performed in conjunction with the Anvi’o taxonomic assignment tool [48] generated 24 bins, accounting for 97.16% of all nucleotides (Tables S2 and S3). All but two of the bins were assigned as being of bacterial origin, confirmed by the presence of 16S/23S ribosomal RNA sequences and the single-copy core gene hits to the GTDB [49] (Fig. 1b; Table S4). It was possible to recover 22 bacterial genomes as near-complete MAGs, ranging in size from 2.9 to 5.5 Mbp (Table S3); a further bin was incomplete and not taxonomically assigned (bin 5; Table S4). Of the 22 complete/near-complete bacterial MAGs, 10 were *Alphaproteobacteria* (including 5 *Rhodobacteraceae*), and 8 were *Gammaproteobacteria* (including 3 *Alteromonadaceae* species) (Table S4). The remaining four genomes were from *Bacteroidia* (all *Flavobacteriales*). Notably, the described bacterial community included many taxa commonly found associated with photosynthetic stramenopile relatives, the diatoms (namely, *Rhodobacteraceae*, *Alteromonadaceae* and *Flavobacteriales*) [64]. Eight of the MAGs (of the genera *Pseudoalteromonas*, *Alteromonas*, *Methylophaga*, *Arenibacter*, *Muricauda*, *Alcanivorax*, *Pararhizobium*, *Hoeflea* and *Thalassospira*) had no species-level taxonomic prediction suggesting potentially novel species, and two bins had taxonomic prediction only to family level (*Crocinitomicaceae* and *Parvibaculaceae*). As an indication of the quality of our metagenomic assembly for these bacteria, 18 of the genomes were >97% complete, and 7 comprised a single contig (Table S3).

The remaining two bins both had 18S/28S rRNA genes that were identified as *I. marina*, alongside the same GC content, and so they were combined to form the *I. marina* MAG (hereinafter referred to as genome) (Tables S3 and S4). The *I. marina* genome was determined to be haploid by calculating k-mer frequencies from *I. marina*-specific reads (Fig. S3). A phylogenomic tree showing the relationship of *I. marina* to other stramenopiles is given in Fig. S4. With a genome of ~68 Mbp (Table S3), the *I. marina* genome is similar in size to many other sequenced stramenopile genomes including *Schizochytrium* (now *Aurantiochytrium*) species [65] and several *Phytophthora* species [65,67], but larger than the diploid diatoms *Thalassiosira pseudonana* (32.4 Mbp [21]) and *P. tricornutum* (27.4 Mbp [68]) and other heterotrophic stramenopiles such as *Cafeteria roenbergensis* BVI (36.3 Mb [69]) or *Blastocystis hominis* Singapore isolate B (18.8 Mb [70]). In fact, genome size varies considerably even within individual stramenopile groups, with an almost 50-fold difference amongst diatoms, from 33 Mb for *Cyclotella nana* to 1.5 Gb in *Thalassiosira tumida* [71] and even ranging from 32 to 295 Mb for the *Phytophthora* genus [72]. These variations are likely due mainly to a higher proportion of repetitive sequences in the larger genomes. In this context, analysis of the repetitive sequence content of the *I. marina* genome using RepeatMasker [73] determined that 20.5% was made up of repeats. This consisted predominantly of simple repeats (5,834,774 bp – 8.57%) and retroelements (2,698,197 bp – 3.96%, including 1.27% large tandem repeat elements), as well as DNA transposons (1.23%).

**Fig. 2.**
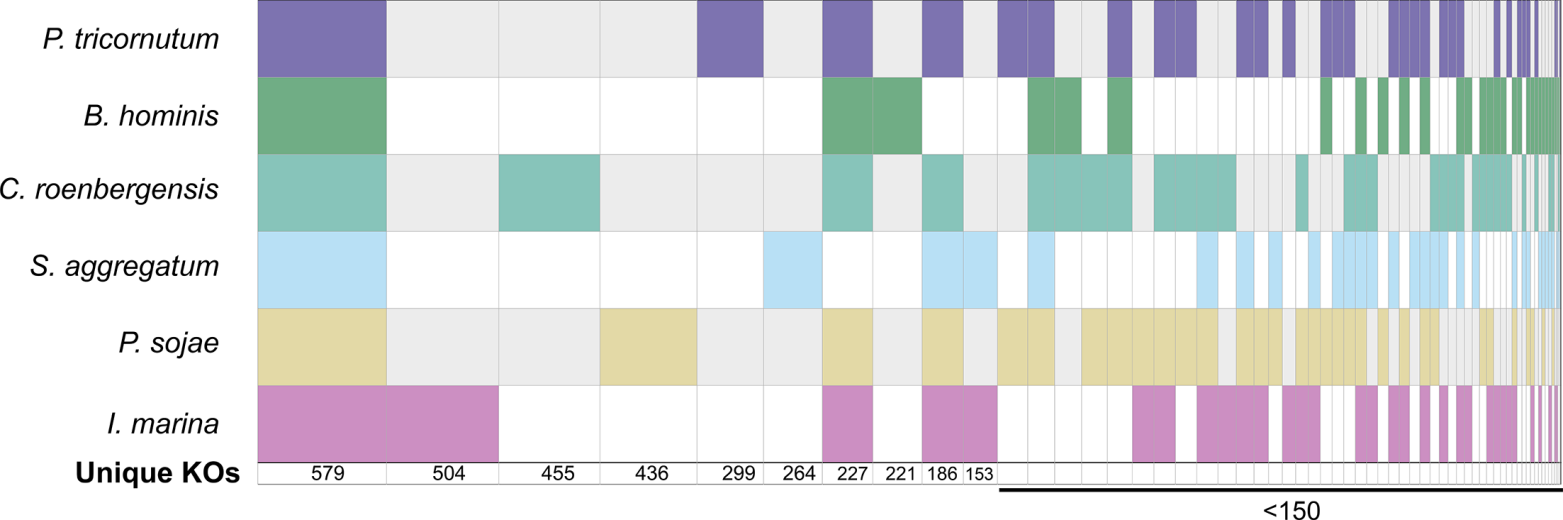
Shared and unique KOs of *I*. *marina* with other stramenopiles. SuperVenn diagram of shared and unique predicted proteins (by KEGG orthology) of *I. marina* with *P. tricornutum*, *P. sojae*, *S. aggregatum*, *C. roenbergensis* and *B. hominis*. These species represent groups across the stramenopiles: Nanomonadea, Bacillariophyceae, Oomycota, Labyrinthula and Bikosia, respectively. Note that sets with fewer than 50 values are not shown.

To investigate the gene complement of *I. marina*, the pipeline shown in Fig. S5 was followed, with the gene-calling software Augustus [74] predicting a total of 20,091 gene models. BUSCO analysis subsequently determined the genome completeness of this predicted gene set to be 93%. A meta-comparison with other sequenced stramenopiles (including 498 accessions from *Tara* [75], MMETSP [76] and Joint Genome Institute) places the *I. marina* genome in the top 10% for genome completeness using BUSCO [77] (Fig. 1c). It should be noted that this analysis compares different types of ‘omics dataset (e.g. genomes of cultured strains versus transcriptomes) but nevertheless gives an indication of the quality of the *I. marina* genome retrieved. Gene annotation analyses revealed that the functional repertoire of *I. marina* is comparable to other sequenced stramenopiles (Fig. S6). The most frequent annotation is ‘Function Unknown’, and many genes have no annotation at all, so only 20% of *I. marina* genes have a known function. Nevertheless, 1,545 genes were annotated with a COG annotation, with post-translational modification (O), translation (J), carbohydrate (G) and amino acid transport/metabolism (E) being the most well-represented categories in both heterotrophic and photosynthetic stramenopiles (Fig. S6). Comparative genomics of shared and unique KOs between the stramenopiles revealed that the largest set of KOs was shared between all species (Fig. 2), likely representing core metabolic functions. However, *I. marina* had the greatest number of unique KOs (504) of the six species, which suggests that it possesses a unique metabolism within the sampled stramenopiles. Amongst these unique proteins, there were 19 lysosome-associated proteins, including 4 cathepsin proteases, as well as 3 branched-chain amino acid transporters.

The presence of a chloroplast of red algal origin in diatoms, which is also shared with many other algal groups including dinoflagellates and haptophytes, had led to the proposal of a single secondary endosymbiosis, the so-called Chromalveolate Hypothesis [78]. However, phylogenomic analysis of nuclear genomes from these different algal groups does not resolve them as monophyletic [79], and instead, it is likely that multiple secondary and tertiary endosymbioses have occurred over evolutionary history [22,23]. Indeed, in searching for evidence of a red-algal-like plastid in the genome of *I. marina*, of 4,208 ‘Protist’ annotated genes, only 74 had unique red algal annotated gene calls, and only 1 was identified as having a predicted protein product with localization to the chloroplast of photosynthetic species (Table S5). This analysis suggests that it is unlikely that the basal MAST-3 lineage of stramenopiles had chloroplasts at any point during its evolutionary history.

### Examining the requirement of *I. marina* for essential vitamins and amino acids

**Fig. 3.**
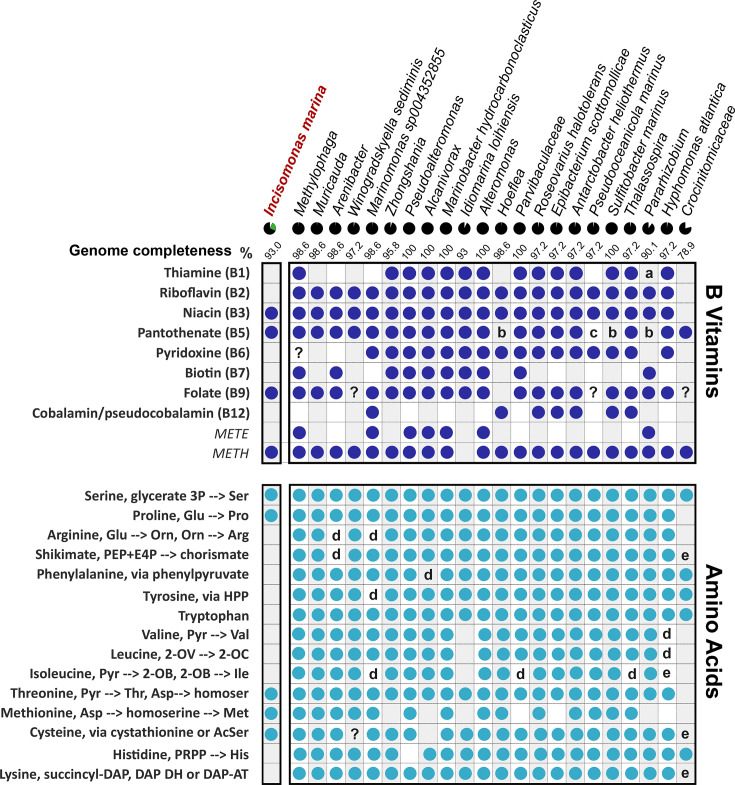
Predicted biosynthetic pathways for B vitamins and amino acids in *I. marina* and its associated bacterial species. Predictions of the *de novo* B vitamin (top) and amino acid (bottom) biosynthetic pathways in each MAG, where a circle represents instances where a pathway is predicted to be complete [or the gene to be present in the case of B_12­_-independent and -dependent methionine synthases (METE/METH, respectively)]. A blank indicates the absence of genes for all or the majority of enzymes, whereas the letters are for one or two missing genes: ‘a’ indicates the enzyme ThiL is not present; ‘b’ = PanB and PanC are missing; and ‘c’ = only PanB is missing. For the amino acid biosynthesis analysis, ‘d’ and ‘e’ indicate where a partial biosynthesis pathway was identified but with one or two enzymes of that pathway missing, respectively, as predicted by KEGG. Ambiguous biosynthetic capability is labelled ‘?’.

To advance knowledge of the nutritional requirements of *I. marina*, we took a more targeted approach. Given the widespread vitamin auxotrophy amongst algal lineages [27,80] and poor understanding of protist vitamin requirements [31], we examined the *I. marina* genome for genes encoding biosynthetic enzymes for B vitamins, which provide essential enzyme cofactors. *I. marina* was predicted to encode complete biosynthesis pathways for B_3_ (niacin), B_5_ (pantothenate) and B_9_ (folate) (Fig. 3, left-hand column) and be auxotrophic for B_1_ (thiamine), B_2_ (riboflavin), B_6_ (pyridoxine) and B_7_ (biotin). The presence of the B_12_-dependent isoform of methionine synthase (*METH*), but not the B_12_-independent isoform (*METE*) [81], suggests that *I. marina* also requires an exogenous supply of B_12_ (cobalamin). Additionally, we identified that *I. marina* encodes both l-galactonolactone dehydrogenase (GLDH) and l-gulonolactone oxidase (GULO) for vitamin C (ascorbate) biosynthesis (Table S6). This was also the case for C. *roenbergensis*, but not *S. aggregatum*, *P. sojae*, *P. tricornutum* or *B. hominis*. In plants and algae, GLDH has functionally replaced GULO, likely due to its ability to decouple ascorbate biosynthesis from H_2_O_2_ production, promoting the role of ascorbate as a photoprotective antioxidant [82]. The selective pressure to retain vitamin C biosynthesis in *I. marina* may have been driven by the inability of bacteria to synthesize this essential cofactor and antioxidant, which could also mitigate oxidative stress resulting from biotic interactions [83]. Thus, whilst *I. marina* clearly requires certain vitamins, contrary to prevailing views on the nutritional dependencies of heterotrophic protists, it has the genes necessary to synthesize several vitamins itself and thus will likely contribute to the cycling of such crucial metabolites in marine microbial communities.

*I. marina* encodes the genes necessary for processing ammonia into organic nitrogen, i.e. via glutamine synthase and glutamine oxoglutarate aminotransferase (GOGAT) (Table 1). We also found genes for enzymes of the urea cycle, previously identified in diatoms [84], and thought to play a role in adaptation to limiting nitrogen conditions. In *P. tricornutum* and *T. pseudonana*, the enzyme that catalyses the first step, carbamoyl phosphate synthase (CPS), is located in the mitochondria [84]. Diatoms also encode a second CPS that lacks an organelle-targeting sequence and is presumed to be involved in cytosolic pyrimidine biosynthesis. In a similar manner, we identified two hits for CPS in *I. marina*, one of which is predicted to be mitochondrially targeted (Table 1). Reciprocal blast of these sequences against the *T. pseudonana* genome yielded both diatom CPS genes (JGI protein IDs: 24248 and 24195). *I. marina* also encodes an arginase, necessary for the production of urea from arginine, as well as ornithine and proline. However, it lacks the capability to synthesize *de novo* many of the protein amino acids (Fig. 3). Whilst there were predicted genes for all enzymes of proline, methionine and cysteine biosynthesis, as well as those derived directly from intermediary metabolism (glycine, alanine, glutamate, glutamine, aspartate, asparagine, serine and threonine), those of pathways for lysine, arginine, the aromatic amino acids (phenylalanine, tyrosine and tryptophan) or the branched-chain amino acids (valine, leucine and isoleucine) were either entirely missing or incomplete. The absence of the shikimate pathway enzymes for the synthesis of chorismate is notable given that *I. marina* encodes the subsequent enzymes to produce folate (B_9_) from chorismate.

**Table 1.** N assimilation and urea cycle enzymes identified in the genome of *I*. *marina*

Process	Enzyme	EC no.	KO no.	*I. marina* protein ID	Predicted targeting category
N assimilation	Glutamine synthetase	6.3.1.2	KO1915	g18223.t1	Other
				g7363.t1	Mitochondrial
				g14366.t1	Other
	GOGAT	1.4.1.13	KO0265	g9516.t1	Mitochondrial
Urea cycle	Arginase	3.5.3.1	KO2965	g12696.t1	Other
	CPS	6.3.4.16	KO1948	g18404.t1	Mitochondrial
		6.3.5.5	KO0370	g8254.t1	Other

The high level of completeness of the genome (93%) suggests that the lack of homologues of genes for both vitamin and amino acid biosynthesis is likely to be correct, although it does not rule out divergent forms of the enzymes or indeed alternative biosynthetic routes [85], which would not be detected in this KO analysis. Nonetheless, it can be concluded that *I. marina* needs to obtain a suite of essential metabolites for normal growth. Identification of a complete urea cycle, as well as branched-chain amino acid transporters, would enable *I. marina* to acquire and detoxify organic compounds obtained through the digestion of bacteria.

To assess the potential for the associated bacterial consortium to provide *I. marina* with its required amino acids and B vitamins, we investigated the metabolic capabilities of the sequenced members. With regard to the B vitamins, just three species (*Pseudoalteromonas* sp., *Alcanivorax* sp. and *M. hydrocarbonoclasticus*) were autotrophic, since they were predicted to produce B_1_–B_9_ and encoded the B_12_-independent METE (Fig. 3, right-hand columns, top panel). In contrast, all the other bacteria appeared to have a requirement for an external source of at least one vitamin. *Crocinitomicaceae* was predicted to produce just one B vitamin, pantothenate (B_5_), although its genome was less than 80% complete. Of species with >90% completion, *Pararhizobium* sp. and *Hoeflea* had just four predicted biosynthesis pathways. Over half the bacteria were missing all four required enzymes for biotin (B_7_) biosynthesis, whereas for those species unable to synthesize pantothenate (B_5_), one lacked just the first committed enzyme, PanB (*Pseudooceanicola*), and three others (*Hoeflea*, *Pararhizobium* and *Sulfitobacter*) lacked both PanB and the last enzyme, PanC.

By comparison, the majority of bacteria encoded the genes necessary for biosynthesis of the protein amino acids (Fig. 3, right-hand columns, bottom panel). As expected, all species synthesizing lysine used the typical eubacterial succinyl-DAP route, rather than the DAP aminotransferase characteristic of eukaryotes, and the majority used the acetylserine route for cysteine biosynthesis, although *Muricauda* and *Arenibacter* encoded enzymes for the alternative route via cystathionine. *Crocinitomicaceae* lacked most enzymes for biosynthesis of proline, arginine, methionine and branched-chain amino acids, as well as the shikimate pathway, but again this might be due to low genome completeness. In contrast, the absence of genes for the biosynthesis of branched-chain protein amino acids in *Hyphomonas atlantica* and *Idiomarina loihiensis* is likely to be the case, since their genomes were 97–93% complete, respectively. The capacity for methionine biosynthesis was observed the least frequently amongst the bacteria, whereas *I. marina* encodes all required enzymes. Together, our analysis provides evidence that *I. marina* can satisfy its requirements for several essential organic nutrients from its co-habiting bacteria and that cross-feeding may also occur between bacterial community members.

### Identification of an *I. marina DSYB* gene for biosynthesis of potent bacterial chemoattractant DMSP

**Fig. 4.**
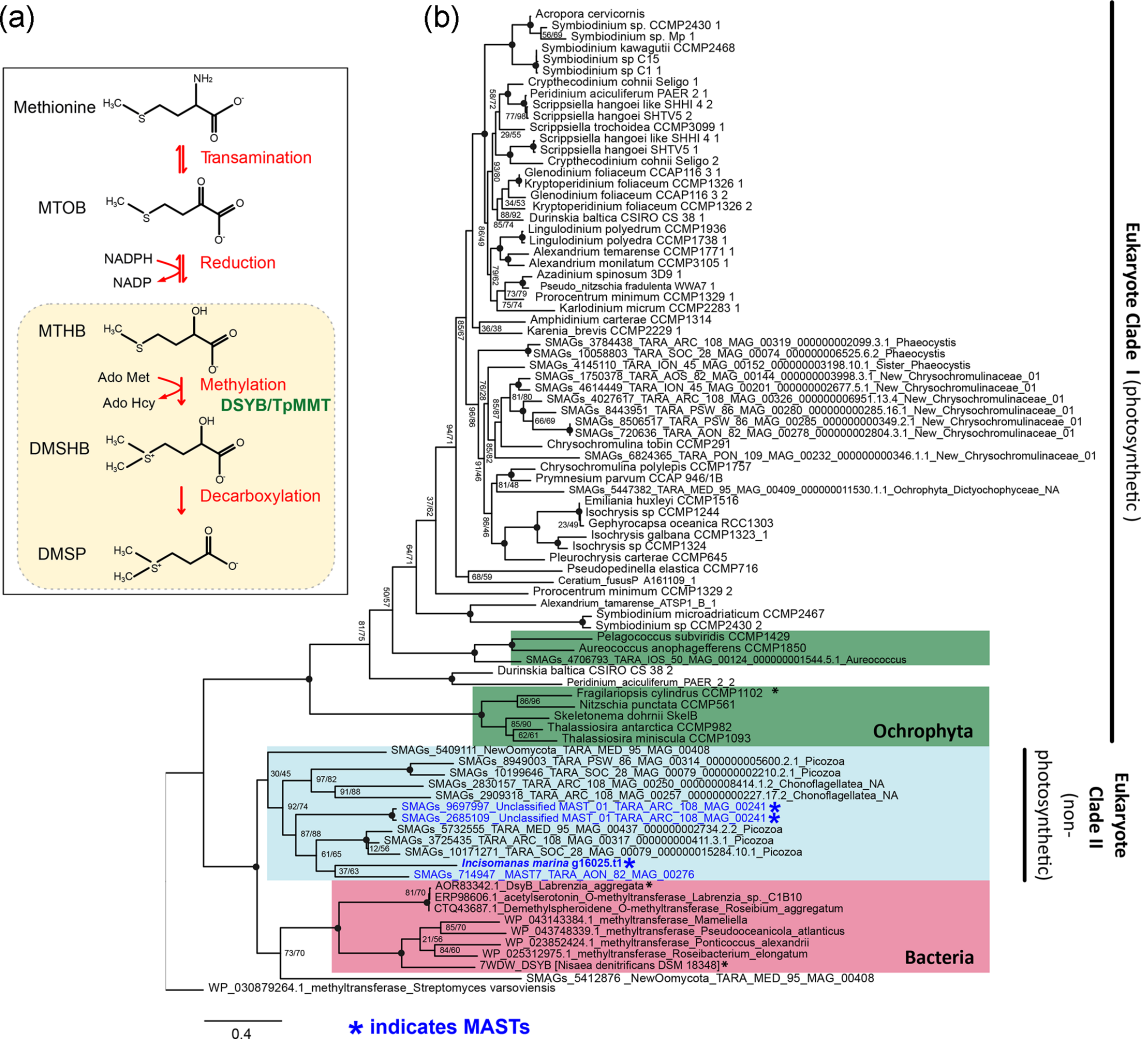
Identification of DMSP biosynthesis enzyme DSYB in *I*. *marina* and several other MASTs. (a) Schematic diagram of the transamination pathway for DMSP biosynthesis from methionine. The key biosynthesis enzyme SAM-dependent MTHB *S*-methyltransferase, DSYB, is indicated in green. DSYB has been identified in heterotrophic bacteria, as well as certain eukaryotic algae [34,35, 53]. The MMT enzyme identified by Kagyama *et al.* (2018) in *T. pseudonana* is an alternative proposed methylation enzyme [93]. MTOB, 4-methylthio-2-oxobutyrate; MTHB, 4-methylthio-2-hydroxybutyrate; DMSHB, 4-dimethylsulphonio-2-hydroxybutyrate. Pathway constructed from [53]. In eukaryotic algae, DSYB enzyme and DMSP synthesis are localized to both chloroplasts and mitochondria (yellow box) [53]. (b) Maximum likelihood gene tree of DSYB protein sequences from bacteria, eukaryotic phytoplankton and new hits identified in this study for *I. marina* and other MASTs (blue). MAST sequences form a new clade of eukaryotic DSYB proteins (‘Eukaryote Clade II’) together with hits from several other heterotrophic eukaryotes (from Picozoa, new Oomycota and choanoflagellates) retrieved from the *Tara Oceans* eukaryotic MAG and SAG databases. The new Eukaryote Clade II is sister to the bacterial clade of DSYBs and sits apart from Eukaryote Clade I that contains eukaryotic phytoplankton DSYB sequences from [53]. Three DSYB enzymes that have been functionally validated are labelled with an ‘*’. The non-DSYB methyltransferase sequence of *Streptomyces varsoviensis* (WP_030879264.1) was used as an outgroup. Groups of related organisms are indicated with coloured boxes. IQTREE was used in model prediction mode to create a maximum likelihood tree using ultrafast bootstrapping and the -altr flag, resulting in two values of node support. Q.pfam+I+R5 was determined to be the best fit model. The final trimmed alignment used for tree construction was 365 amino acids in length. Node support values were removed above 90/90 and are indicated with a filled black circle.

DMSP is an abundant marine organosulphur compound with important roles in stress protection and signalling [32]. Produced by many bacteria [34] and eukaryotic phytoplankton [53], DMSP gives rise to the climate-active gas, dimethylsulphide. DMSP is typically produced through the transamination pathway [34], via *S*-adenosylmethionine (SAM)-dependent 4-methylthio-2-hydroxybutyrate (MTHB) *S*-methyltransferase, encoded by the DSYB/DsyB gene [34,53] (Fig. 4a), although an alternative algal biosynthesis enzyme, DSYE, has recently been identified [35].

**Fig. 5.**
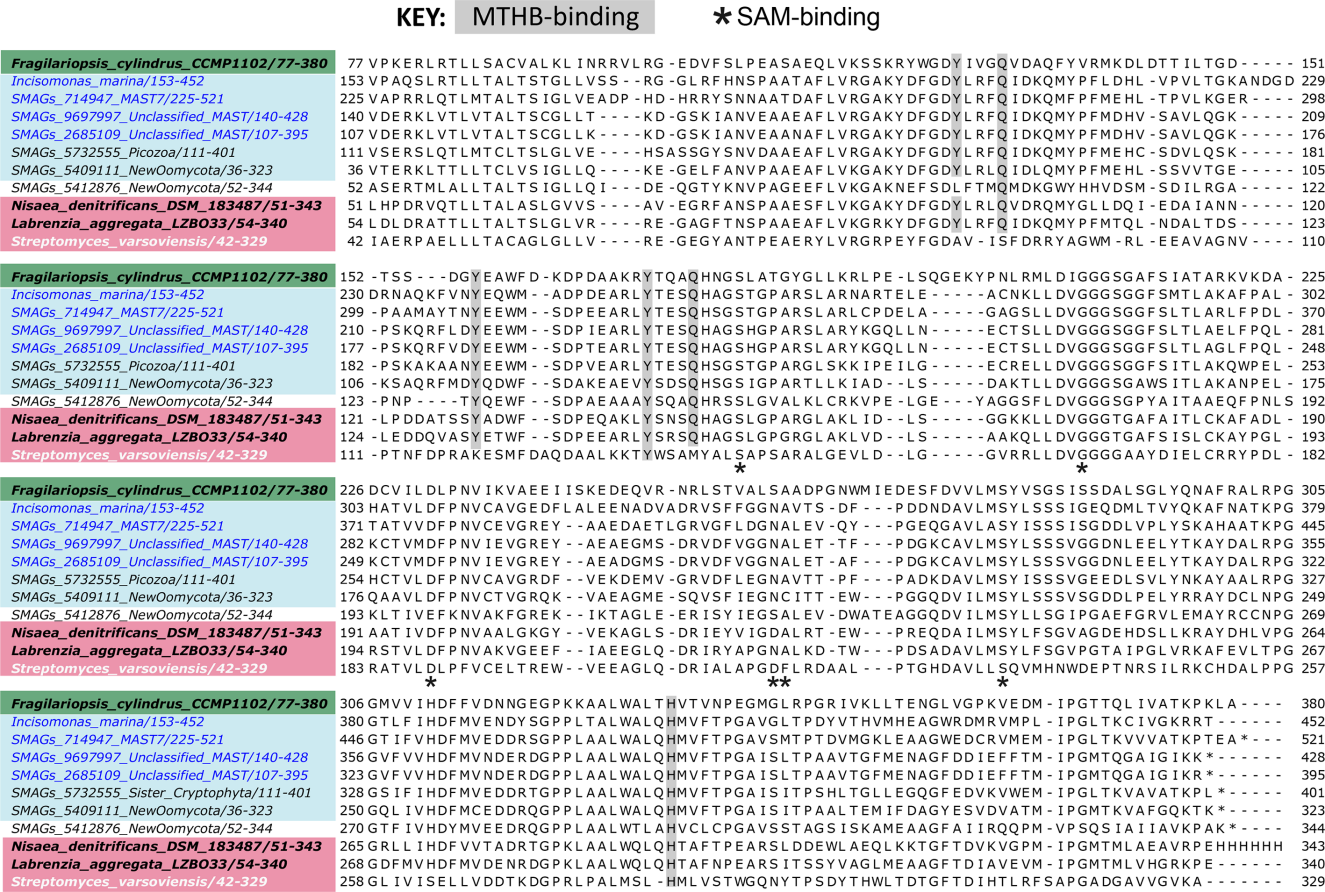
Multiple sequence alignment of bacterial and eukaryotic DsyB/DSYB protein sequences. DsyB protein sequences that have been cloned and functionally characterized from the bacteria *Nisaea denitrificans* (NCBI identifier: 07WDW_A) [87], *Labrenzia aggregata LZB033* (NCBI identifier: AOR83342) [34] and the diatom *F. cylindrus* (JGI protein ID: 238045) [53] are highlighted in bold. The sequence of a ‘non-DsyB’ methyltransferase enzyme from *S. varsoviensis* (NCBI identifier: WP_030879264.1) incapable of DMSP biosynthesis is white. Sequences from *I. marina* and representatives from ‘Eukaryote Clade II’ shown in Fig. 4(b) are also included, as well as both ‘New Oomycota’ sequences. Taxa are coloured according to their phylogenetic grouping from the maximum likelihood tree in Fig. 4(b). Residues involved in MTHB binding and SAM binding are labelled as indicated.

In photosynthetic algae, DSYB is localized to both chloroplasts and mitochondria [53]. Previous searches of heterotrophic stramenopiles [including Labyrinthulea *Aplanochytrium* (sp. PBS07 and *A. stocchinoi* GSBS06), *Aurantiochytrium limacinum* ATCCMYA1381 and *S. aggregatum* ATCC28209] did not yield hits for *DSYB* [53]. Nor did we find the gene in *B. hominis* Singapore isolate B (sub-type 7) or *C. roenbergensis* BVI. However, querying the *I. marina* genome with a diatom sequence (*Fragilariopsis cylindrus*, protein 238045) [53] yielded a robust hit (e-value: 1.5e^−22^; percentage identity: 42%). Moreover, a mitochondrial transit peptide was identified. To examine the presence of putative DSYB sequences in other MASTs, we used the *I. marina* sequence to query the *Tara Oceans* eukaryotic metagenome and SAG database (EUK_SMAG) [52]. We retrieved three robust hits for MASTs (all hits with an e-value cut-off below 1e^−70^ are shown in Table S7). A further two were obtained from a MAG taxonomically assigned as ‘New Oomycota’ (i.e. heterotrophic stramenopile taxa) [75]. Our search also yielded sequences from choanoflagellates and Picozoa, as well as eukaryotic phytoplankton, with many predicted to be mitochondrially localized (Table S7). A further two hits for heterotrophic Picozoa were obtained by mining additional SAG databases [86] (F_COSAG_04_NODE_10F_COSAG02 and F_COSAG04_NODE_1660). Construction of a maximum likelihood tree revealed that the MAST sequences (labelled blue) grouped together with robust support (Fig. 4b), along with sequences from eukaryote taxa, in all cases predicted to be heterotrophic (Table S7) [75]. This new clade of heterotrophic eukaryotic DSYBs (‘Eukaryote Clade II’; Fig. 4b) groups separately from the established eukaryotic clade (‘Clade I’), containing algal DSYBs [53], and is instead sister to the bacterial clade [34,87]. A multiple sequence alignment of functionally verified DSYB/DsyB enzymes demonstrates that the MAST sequences have key residues experimentally determined to be necessary for DSYB/DsyB function, including for MTHB and SAM binding (Fig. 5), indicating that they are likely functional.

**Fig. 6.**
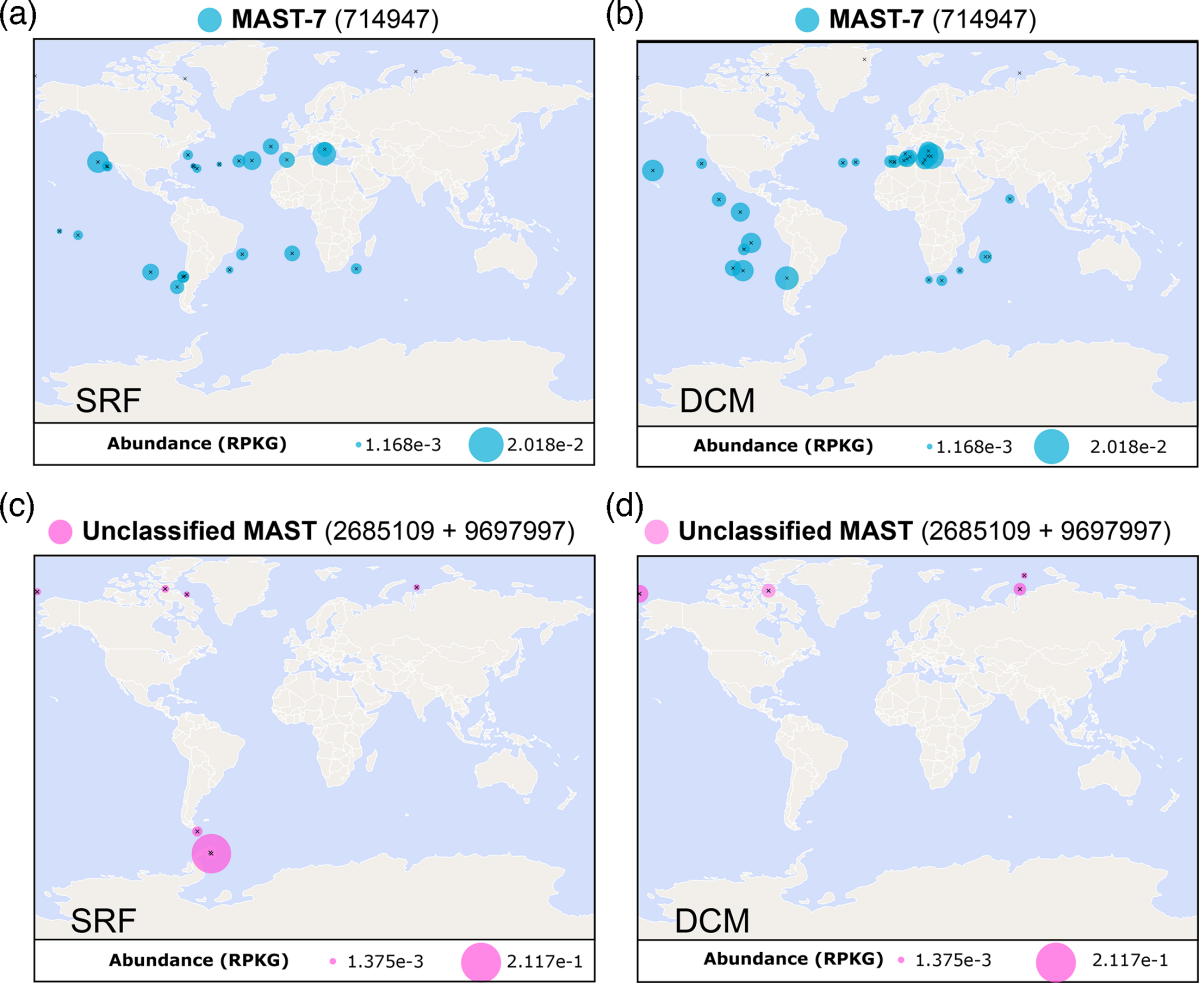
Global abundance of MAST DSYB in marine systems. Bubble plots showing abundance (as a percentage of total reads) of ‘MAST-7 hit’ SMAG_714947 in SRF (**a**) and the DCM (**b**). Equivalent plots are also shown for ‘unclassified MAST’ hits (SMAG_2685109 and SMAG_9697997) (**c, d**). Data were compiled through querying eukaryotic MAGs and SAGs via the Ocean Gene Atlas portal [52].

Plotting the distribution of the MAST SAG and MAG hits revealed a cosmopolitan distribution in marine systems globally of the DSYB MAST-7 hit (Fig. 6a, b), whereas the ‘Unclassified MAST’ sequences showed a more confined biogeography (Fig. 6c, d). Nevertheless, the identification of convincing DSYB sequences in MAST lineages, as well as from other heterotrophic eukaryotes, suggests that the contribution of such protists to global DMSP cycling is likely more significant than previously recognized. Given that DMSP is a potent chemoattractant and important nutrient source for many marine bacteria [33], DMSP biosynthesis could thus enable MASTs to attract bacteria. Shao *et al.* [88] describe a four-enzyme pathway in marine *Roseobacter* spp. for DMSP degradation*,* resulting in the production of acetylaldehyde and methanethiol, used as carbon and sulphur sources, respectively [88]. We searched for the genes encoding the enzymes, *dmdA*, *dmdB*, *dmdC* and *dmdD/acuH*, in the metagenome (Table S8). Although no single organism had the whole pathway, all enzymes were present in at least two bins, indicating the possibility of metabolic cooperation within the consortium for DMSP degradation, a widespread concept underpinning the stability of such consortia [89,90]. Interestingly, the *dmdD/acuH* gene was found in the *I. marina* genome, suggesting that it too might play a role in the process.

## Conclusion

Across the eukaryotic tree of life, there is considerable bias in genomic sequencing [2]. This is acutely apparent in the stramenopile group, which displays an enormous range of habitat, trophic and cell structural diversity, and yet sequencing efforts focus mainly on photosynthetic lineages and terrestrial oomycetes [2,21, 68, 91]. In contrast, the basal lineages are poorly understood and understudied. This is despite the fact that MASTs are likely major players in ocean carbon cycling and the marine microbial loop [9] with recent work identifying MAST-3s as grazers of *Prochlorococcus,* one of the most globally abundant phytoplankton taxa [92]. Our study provides important evidence that nutritional demands for certain B vitamins and amino acids may well be key factors driving such predator–prey interactions. However, *I. marina* encodes genes necessary to synthesize several vitamins itself (vitamins C, B_3_, B_5_ and B_9_). Notably, our metabolic pathway analysis also reveals that no single member of the *I. marina* consortium is capable of synthesizing all the organic nutrients examined. Metagenomic analysis of the ‘meta-metabolome’ thus suggests that cross-exchange of nutrients is possible between members of this community, which has been maintained stably since its isolation from environmental samples. Although the cultivation medium for the *I. marina* consortium is undefined, containing both soil extract and a barley grain, our findings provide the basis for further dissection of the growth requirements, potentially enabling laboratory cultivation of more MAST species.

Further, our work reveals unexpected metabolic attributes of MASTs. Whilst not previously found in other heterotrophic stramenopile genomes, we identified the DMSP biosynthesis gene *DSYB* in *I. marina*, as well as environmental MAST MAGs and SAGs. These findings raise important questions regarding the evolution of DMSP biosynthesis in eukaryotes and highlight a role for heterotrophic stramenopiles in global DMSP production and sulphur cycling. Finally, our study also indicates that key metabolic innovations of ancestral heterotrophic stramenopiles (urea cycle, GLDH pathway for vitamin C biosynthesis and DMSP biosynthesis) may have provided important foundations for the evolution of some of the most successful groups of phototrophic organisms on the planet and illustrates the need to extend investigation of the attributes of these enigmatic organisms.

## Supplementary material

10.1099/mgen.0.001510 Uncited Supplementary Material 1.

## Abbreviations

ASWP: Artificial Seawater for Protozoa
CPS: carbamoyl phosphate synthase
DCM: deep chlorophyll maxima
DMSP: dimethylsulphoniopropionate
GLDH: l-galactonolactone dehydrogenase
GOGAT: glutamine oxoglutarate aminotransferase
GTDB: Genome Taxonomy Database
GULO: l-gulonolactone oxidase
HMMs: Hidden Markov Models
KO: KEGG orthologue
MAGs: metagenome-assembled genomes
MASTs: MArine STramenopiles
MMETSP: Marine Microbial Eukaryote Transcriptome Sequencing Project
MTHB: 4-methylthio-2-hydroxybutyrate
ONT: Oxford Nanopore Technologies
SAG: Single-cell Assembled Genome
SAM: *S*-adenosylmethionine
SEM: scanning electron microscopy
SRF: surface waters
