# Supplementary material for: Metagenomics of the MAST-3 stramenopile, Incisomonas, and its associated microbiome reveals unexpected metabolic attributes and extensive nutrient dependencies
Source: Microb Genom. 2025 Nov 13;11(11):001510. doi: 10.1099/mgen.0.001510 (PMC12614181; doi:10.1099/mgen.0.001510)
Supplement: Uncited Supplementary Material 1. [file mgen-11-01510-s001.pdf]

Metagenomics of the MAST-3 stramenopile, *Incisomonas,* and its associated microbiome reveals unexpected metabolic attributes and extensive nutrient dependencies

Dominic E Absolon, Victoria LN Jackson, Adam Monier, Alison G Smith & Katherine E Helliwell

<u>Supplementary Figures and Tables</u>

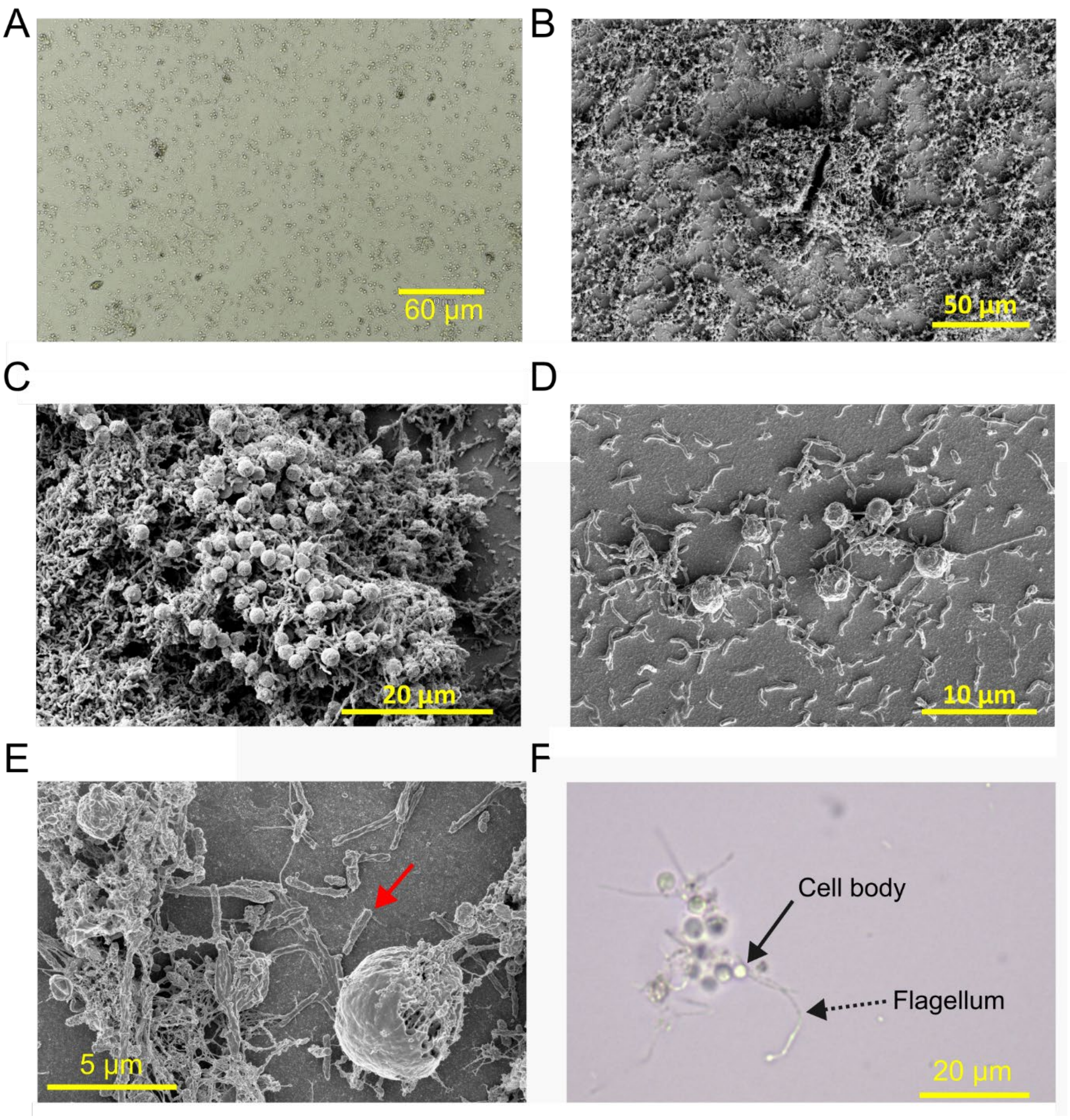

Figure S1. Imaging of the *Incisomonas marina* CCAP 977/1 culture. A. Light microscopy image of *I. marina* and bacteria (40× magnification). **B-D**. Scanning Electron Microscopy (SEM) images of *I. marina* and associated bacteria displaying benthic growth and clumping behaviour. **E**. SEM image of dead and decaying *I. marina* cells (e.g., bottom right quadrant) with associated bacteria in close proximity (red arrow). **F.** Oil immersion 100× magnification light microscopy image of *I. marina* showing typical clumps that form in the medium. The cell body and flagellum *of I. marina* are annotated with arrows.

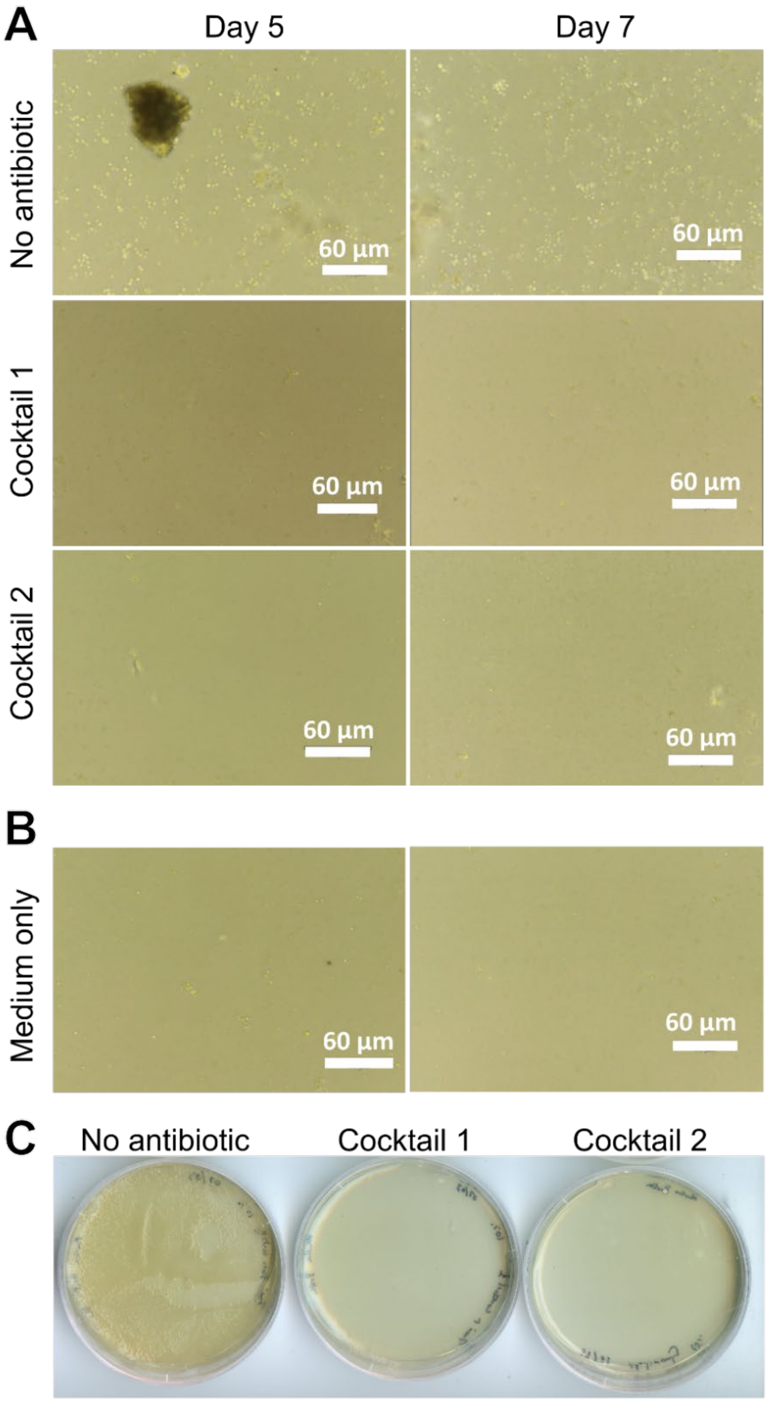

Figure S2. Treatment of *Incisomonas marina* cultures with antibiotics eliminated growth of *I. marina* and bacteria. A. Light microscopy images of *I. marina* cultures after 5- and 7-days treatment with two different antibiotic cocktails (**Table S1**), compared to the no antibiotic control. **B.** Images taken of 'media only' controls containing artificial seawater for protozoa (ASWP) medium + grain i.e., without inoculation with the *I. marina* consortium. **C.** Images of marine broth plates inoculated with samples taken of *I. marina* cultures treated with antibiotic cocktails compared to control.

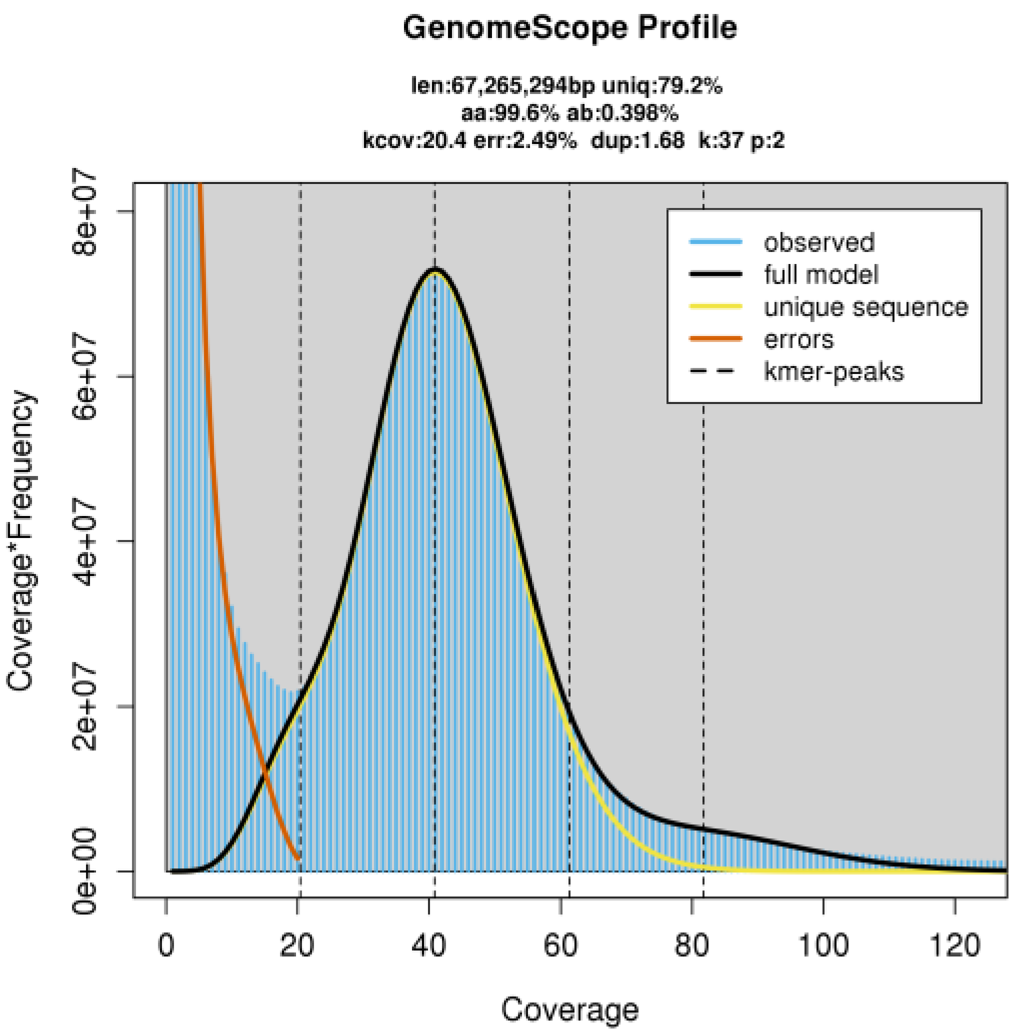


```
GenomeScope version 2.0

p = 2
k = 37

property                    min              max
Homozygous (aa)             99.5901%         99.6141%
Heterozygous (ab)           0.3859%          0.40985%
Genome Haploid Length       67,125,052 bp    67,265,294 bp
Genome Repeat Length        13,953,246 bp    13,982,398 bp
Genome Unique Length        53,171,806 bp    53,282,896 bp
Model Fit                   85.3674%         99.0997%
Read Error Rate             2.48741%         2.48741%
```

Figure S3. Plot of k-mer frequencies in the reads used for the *Incisomonas marina* MAG. K-mer size of 37, as predicted using Genomescope2 (Methods). The single peak demonstrates the haploid nature of the genome. Plot generated by Genomescope2 [1].

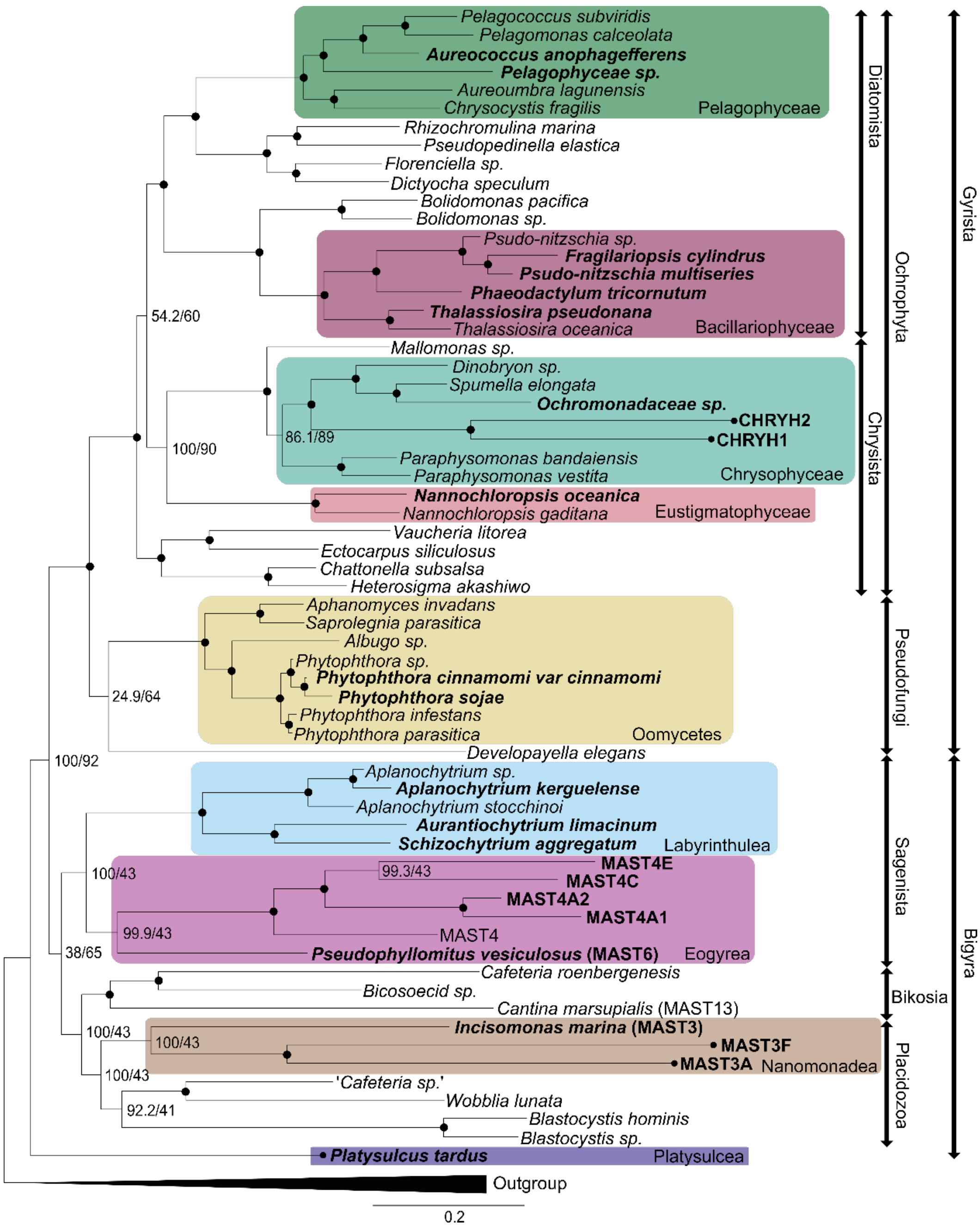

Figure S4. Phylogenomic tree of the Stramenopila. The tree was created using a concatenated alignment of 120 genes with columns removed where 20% of sites were gaps. Gene alignments from Thakur et al., (2019) [2] were used with additional species of interest included to increase taxon sampling. IQtree was used in model prediction mode to create a maximum likelihood tree using ultrafast bootstrapping and the -altr flag, resulting in two values of node support. The model used was VT+F+I+G4. Taxonomic ranking on the right of the tree reflects those of Thakur et al., 2019 [2]. The outgroup includes species from the Rhizaria and Alveolata. Nodes with support values 100/100 are indicated with a filled black circle.

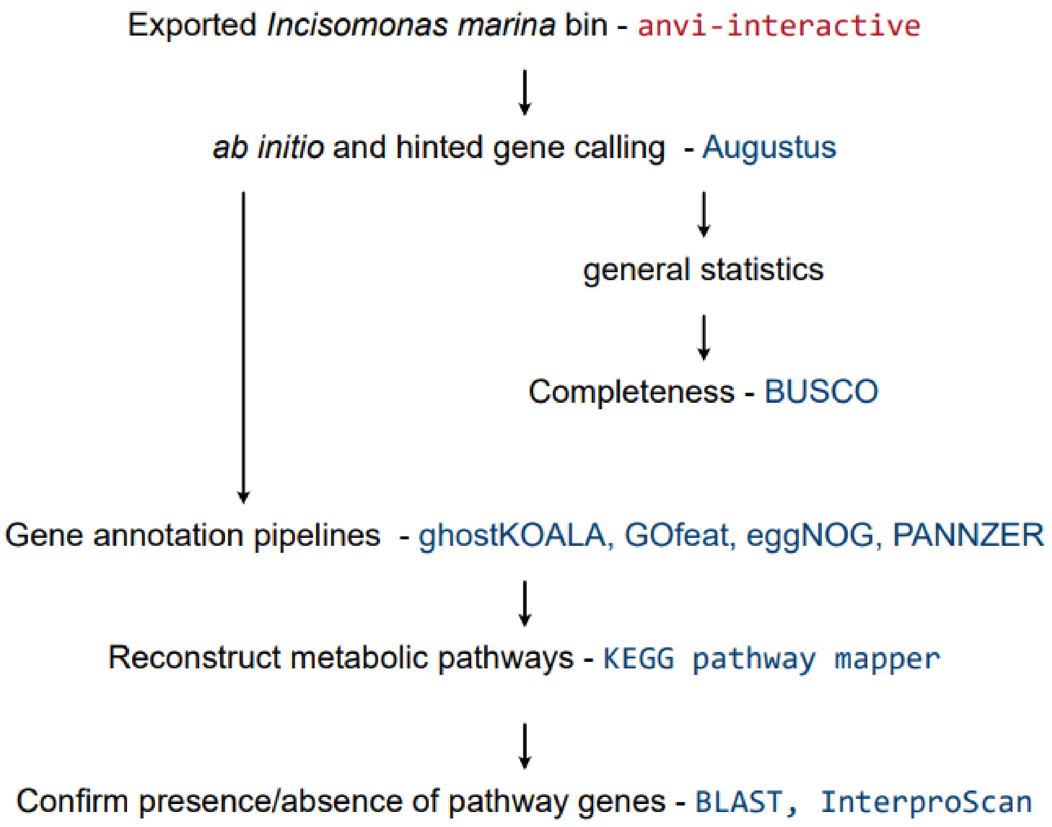

Figure S5. Schematic of the downstream analysis of the *Incisomonas marina* MAG. This includes gene calling, completeness estimation, annotation and pathway analysis.

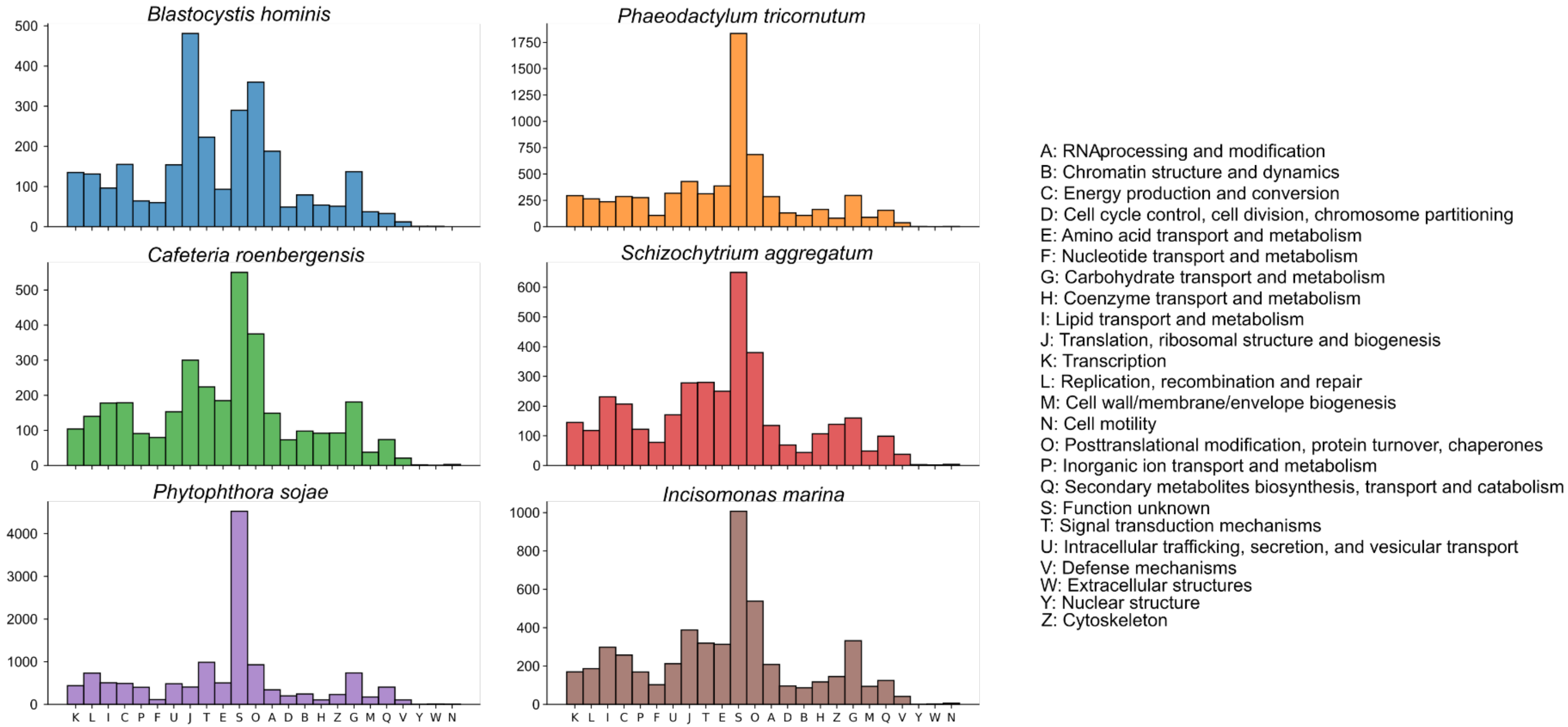

Figures S6. **Assignments of Clusters of Orthologous Genes (COG) categories across the annotated genes of six stramenopiles**. The relative proportions of orthologous genes in the *I. marina* genome were compared to those in 5 other representative stramenopile species to identify common features. These genomes were obtained from the Joint Genome Institute (JGI) website, accessed via the JGI Gold Project ID (Gp) as follows: *Cafeteria roenbergensis* (Gp0616755; [3]), *Blastocystis hominis* (Gp0002361; [4]), *Phaeodactylum tricornutum* (Gp0002884; [5]),

Phytophthora sojae (Gp0017785; [6]) and *Schizochytrium aggregatum* (Gp0002963; [7]). At this macro-scale of annotation, each organism displays a relatively similar functional landscape.

Table S1. Summary of antibiotic cocktails used to treat *Incisomonas marina* cultures. Concentrations successfully employed in the literature to treat cultures of other stramenopile taxa are also given: [a][8]; [b][9]; [c][10]; [d][11].

**Antibiotic**	**Cocktail 1** (µg/ml) (this study)	**Cocktail 2** (µg/ml) (this study)	**Concentrations used in the literature against other Stramenopile taxa** (µg/ml)	**Taxa**
Rifampicin	10	-	300[a]	Thraustrochytrids
Streptomycin	100	30	1600[b]	Diatoms
Ampicillin	100	50	700[c]	Diatoms
Gentamycin	100	-	50-100[d]	Diatoms
Neomycin	-	60	400[b]	Diatoms
Kanamycin	-	50	>250[d]	Diatoms
Chloramphenicol	-	25	400[b]	Diatoms

Table S2. Summary statistics (1 of 2) of metagenome bins of *Incisomonas marina* and bacterial isolates. The metagenome was assembled using Oxford Nanopore long-read data and polished using Derrelle et al., (2016) Illumina short-read data [12] with Flye and Pilon respectively. Supervised binning was performed using the Anvi'o interactive interface [13].

Bin No.	Variability	Relative abundance	Std coverage	Mean coverage, Q2Q3	Mean coverage	Max normalised ratio	Abundance	Bins % recruitment	Detection %
1	51.26	1	8.88	85.66	85.48	1	0.90	36.83	99.72
2	50.00	1	30.61	426.92	424.67	1	4.49	11.18	99.99
3	32.73	1	5.27	57.19	57.14	1	0.60	1.65	100.00
4	2.05	1	2.83	19.06	19.07	1	0.20	0.35	100.00
5	27.53	1	18.68	193.69	192.72	1	2.04	3.49	99.61
6	26.08	1	9.94	153.42	152.93	1	1.62	3.79	100.00
7	0.14	1	2.48	4.80	5.15	1	0.05	0.10	99.85
8	35.68	1	5.52	61.74	61.68	1	0.65	2.16	100.00
9	28.52	1	24.68	554.37	551.61	1	5.83	13.94	100.00
10	0.60	1	2.02	9.32	9.36	1	0.10	0.25	100.00
11	18.33	1	3.93	35.26	35.25	1	0.37	0.80	100.00
12	44.31	1	9.59	150.10	149.88	1	1.58	3.55	99.82
13	45.63	1	6.62	87.08	86.96	1	0.92	2.28	100.00
14	7.19	1	3.33	24.37	24.33	1	0.26	0.72	100.00
15	48.14	1	13.63	234.79	234.19	1	2.47	6.64	100.00
16	51.77	1	7.06	86.61	86.54	1	0.91	2.78	100.00
17	0.55	1	30.05	6.88	16.83	1	0.18	0.35	99.92
18	131.61	1	7.72	74.34	73.84	1	0.78	2.39	100.00
19	6.92	1	2.64	18.50	18.53	1	0.20	0.42	100.00
20	45.23	1	8.49	130.26	129.93	1	1.37	3.97	100.00
21	2.37	1	2.09	9.48	9.53	1	0.10	0.18	99.91
22	0.64	1	1.75	6.58	6.60	1	0.07	0.18	99.91
23	3.61	1	2.38	13.98	14.02	1	0.15	0.39	99.99
24	1.79	1	2.52	14.57	14.59	1	0.15	0.35	100.00
Un-binned	-	-	-	-	-	-	-	1.27	-

Table S3. Summary statistics (2 of 2) of metagenome bins of *Incisomonas marina* and bacterial isolates. The metagenome was assembled using Oxford Nanopore long-read data and polished using Derrelle et al., (2016) Illumina short-read data [12] with Flye and Pilon respectively. Supervised binning was performed using the Anvi'o interactive interface [13]. *Percent completion for bin 1 using the standard Anvi'o eukaryotic BUSCO library. However, when the stramenopile library was used, completion was 93.0%.

Bin No.	Total length (bp)	Number contigs	N50	GC content	Percent completion	Percent redundancy
1	68,357,064	213	1215950	52.5	68.7*	16.9
2	4,108,619	5	4006367	40.9	100	5.6
3	4,510,811	3	4487859	43.3	100	2.8
4	2,913,340	1	2913340	44.8	98.6	4.2
5	2,900,267	63	94070	39.4	0	0
6	3,899,295	2	3863077	33.4	97.2	16.9
7	3,085,312	21	266584	39.0	78.9	15.5
8	5,505,433	2	5469367	39.7	98.6	2.8
9	3,945,902	1	3945902	41.6	98.6	4.2
10	4,219,735	4	2482457	49.6	95.8	7.0
11	3,566,829	1	3566829	63.6	97.2	2.8
12	3,706,920	3	3586063	57.0	100	2.8
13	4,096,611	1	4096611	57.4	100	0
14	4,658,551	1	4658551	61.5	100	0
15	4,425,738	1	4425738	61.0	97.2	8.5
16	5,035,835	4	4700859	61.7	97.2	1.4
17	4,175,398	17	605885	60.5	90.1	9.9
18	5,058,777	2	4789643	59.1	98.6	0
19	3,516,122	1	3516122	58.0	97.2	7.0
20	4,785,063	2	4571768	53.5	97.2	0
21	2,943,871	8	842461	46.9	93.0	5.6
22	4,331,361	10	543885	44.9	98.6	9.9
23	4,324,906	2	4265747	66.8	97.2	7.0
24	3,704,244	1	3704244	62.7	100	0

Table S4. Taxonomic predictions of each bin as predicted by Anvi'o taxonomic prediction. There is no taxonomic prediction for bin 1 as this is the eukaryotic bin belonging to *I. marina,* indicated by the identified locations of 18S/28S rRNA genes. Those indicated with an * are species that were identified from bacterial colonies grown from the consortium and confirmed with PCR of the 16S rRNA genes.

Bin No.	Domain	Phylum	Class	Order	Family	Genus	Species
1	-	-	-	-	-	-	-
2	*Bacteria*	*Proteobacteria*	*Gammaproteobacteria*	*Enterobacterales*	*Alteromonadaceae*	*Pseudoalteromonas*	-
3	*Bacteria*	*Proteobacteria*	*Gammaproteobacteria*	*Enterobacterales*	*Alteromonadaceae*	*Alteromonas*	-
4	*Bacteria*	*Proteobacteria*	*Gammaproteobacteria*	*Nitrosococcales*	*Methylophagaceae*	*Methylophaga*	-
5	-	-	-	-	-	-	-
6	*Bacteria*	*Bacteroidota*	*Bacteroidia*	*Flavobacteriales*	*Flavobacteriaceae*	*Winogradskyella*	*Winogradskyella sediminis**
7	*Bacteria*	*Bacteroidota*	*Bacteroidia*	*Flavobacteriales*	*Crocinitomicaceae*	-	-
8	*Bacteria*	*Bacteroidota*	*Bacteroidia*	*Flavobacteriales*	*Flavobacteriaceae*	*Arenibacter*	-
9	*Bacteria*	*Bacteroidota*	*Bacteroidia*	*Flavobacteriales*	*Flavobacteriaceae*	*Muricauda*	-
10	*Bacteria*	*Proteobacteria*	*Gammaproteobacteria*	*Pseudomonadales*	*Spongiibacteraceae*	*Zhongshania*	*Zhongshania sp002915595*
11	*Bacteria*	*Proteobacteria*	*Alphaproteobacteria*	*Rhodobacterales*	*Rhodobacteraceae*	*Roseovarius*	*Roseovarius halotolerans*
12	*Bacteria*	*Proteobacteria*	*Alphaproteobacteria*	*Rhodobacterales*	*Rhodobacteraceae*	*Sulfitobacter*	*Sulfitobacter marinus*
13	*Bacteria*	*Proteobacteria*	*Gammaproteobacteria*	*Pseudomonadales*	*Oleiphilaceae*	*Marinobacter*	*Marinobacter hydrocarbonoclasticus**
14	*Bacteria*	*Proteobacteria*	*Gammaproteobacteria*	*Pseudomonadales*	*Alcanivoracaceae*	*Alcanivorax*	-
15	*Bacteria*	*Proteobacteria*	*Alphaproteobacteria*	*Rhodobacterales*	*Rhodobacteraceae*	*Epibacterium*	*Epibacterium scottomollicae*
16	*Bacteria*	*Proteobacteria*	*Alphaproteobacteria*	*Rhodobacterales*	*Rhodobacteraceae*	*Antarctobacter*	*Antarctobacter heliothermus*
17	*Bacteria*	*Proteobacteria*	*Alphaproteobacteria*	*Rhizobiales*	*Rhizobiaceae*	*Pararhizobium*	-
18	*Bacteria*	*Proteobacteria*	*Alphaproteobacteria*	*Rhizobiales*	*Rhizobiaceae*	*Hoeflea*	-
19	*Bacteria*	*Proteobacteria*	*Alphaproteobacteria*	*Caulobacterales*	*Hyphomonadaceae*	*Hyphomonas*	*Hyphomonas atlantica*
20	*Bacteria*	*Proteobacteria*	*Alphaproteobacteria*	*Rhodospirillales*	*Thalassospiraceae*	*Thalassospira*	-
21	*Bacteria*	*Proteobacteria*	*Gammaproteobacteria*	*Enterobacterales*	*Alteromonadaceae*	*Idiomarina*	*Idiomarina loihiensis*
22	*Bacteria*	*Proteobacteria*	*Gammaproteobacteria*	*Pseudomonadales*	*Marinomonadaceae*	*Marinomonas*	*Marinomonas sp004352855*
23	*Bacteria*	*Proteobacteria*	*Alphaproteobacteria*	*Rhodobacterales*	*Rhodobacteraceae*	*Pseudooceanicola*	*Pseudooceanicola marinus**
24	*Bacteria*	*Proteobacteria*	*Alphaproteobacteria*	*Parvibaculales*	*Parvibaculaceae*	-	-

Table S5. Red algal annotated genes in *Incisomonas marina*, as annotated by ghostKOALA [14]. Evidence for plastid associated red algal genes were derived from https://www.arabidopsis.org/servlets/TairObject?type=locus&name=AT1G80380. Predicted subcellular localisation of corresponding *I. marina* genes as inferred by HECTAR [15] are also given. Key: n = not predicted.

Gene ID	KO number	Description	Predicted Cp/Mt localisation of *I. marina* predicted protein via HECTAR	Plastid linked?
**g44.t1**	K08288	PRKCSH; protein kinase C substrate 80K-H	n	n
**g504.t1**	K19612	PDE12; 2',5'-phosphodiesterase [EC:3.1.13.4 3.1.4.-]	n	Mitochondrial
**g569.t1**	K18156	ATP23, XRCC6BP1; mitochondrial inner membrane protease ATP23 [EC:3.4.24.-]	n	Mitochondrial
**g1394.t1**	K01875	SARS, serS; seryl-tRNA synthetase [EC:6.1.1.11]	n	Mitochondrial
**g1556.t1**	K00942	gmk, GUK1; guanylate kinase [EC:2.7.4.8]	n	n
**g1679.t1**	K05917	CYP51; sterol 14alpha-demethylase [EC:1.14.14.154 1.14.15.36]	n	n
**g1705.t1**	K07305	msrB; peptide-methionine (R)-S-oxide reductase [EC:1.8.4.12]	n	n
**g1872.t1**	K18156	ATP23, XRCC6BP1; mitochondrial inner membrane protease ATP23 [EC:3.4.24.-]	n	Mitochondrial
**g1888.t1**	K00939	adk, AK; adenylate kinase [EC:2.7.4.3]	n	n
**g1969.t1**	K08592	SENP1; sentrin-specific protease 1 [EC:3.4.22.68]	n	n
**g2756.t1**	K20347	TMED2, EMP24; p24 family protein beta-1	n	n
**g3076.t1**	K12821	PRPF40, PRP40; pre-mRNA-processing factor 40	n	n
**g3633.t1**	K08288	PRKCSH; protein kinase C substrate 80K-H	n	n
**g3942.t1**	K08592	SENP1; sentrin-specific protease 1 [EC:3.4.22.68]	n	n
**g4827.t1**	K11498	CENPE; centromeric protein E	n	n
**g4368.t1**	K08796	BRSK; BR serine/threonine kinase [EC:2.7.11.1]	n	n
**g4619.t1**	K00939	adk, AK; adenylate kinase [EC:2.7.4.3]	n	n
**g4872.t1**	K11498	CENPE; centromeric protein E	n	n
**g4885.t1**	K08592	SENP1; sentrin-specific protease 1 [EC:3.4.22.68]	n	n
**g5477.t1**	K11498	CENPE; centromeric protein E	n	n
**g5867.t1**	K00939	adk, AK; adenylate kinase [EC:2.7.4.3]	n	n
**g6309.t1**	K08288	PRKCSH; protein kinase C substrate 80K-H	n	n
**g7000.t1**	K20347	TMED2, EMP24; p24 family protein beta-1	n	n
**g7257.t1**	K00939	adk, AK; adenylate kinase [EC:2.7.4.3]	n	n

**g7265.t1**	K13703	ABHD11; abhydrolase domain-containing protein 11	Mitochondrial	n
**g8047.t1**	K12821	PRPF40, PRP40; pre-mRNA-processing factor 40	n	n
**g8708.t1**	K08592	SENP1; sentrin-specific protease 1 [EC:3.4.22.68]	n	n
**g9448.t1**	K00939	adk, AK; adenylate kinase [EC:2.7.4.3]	n	n
**g9925.t1**	K08592	SENP1; sentrin-specific protease 1 [EC:3.4.22.68]	n	n
**g10230.t1**	K17777	TIM9; mitochondrial import inner membrane translocase subunit TIM9	n	Mitochondrial
**g10403.t1**	K12821	PRPF40, PRP40; pre-mRNA-processing factor 40	n	n
**g10658.t1**	K00939	adk, AK; adenylate kinase [EC:2.7.4.3]	n	n
**g11052.t1**	K03469	rnhA, RNASEH1; ribonuclease HI [EC:3.1.26.4]	n	n
**g11660.t1**	K12821	PRPF40, PRP40; pre-mRNA-processing factor 40	n	n
**g11580.t1**	K00939	adk, AK; adenylate kinase [EC:2.7.4.3]	n	n
**g11705.t1**	K08592	SENP1; sentrin-specific protease 1 [EC:3.4.22.68]	n	n
**g12317.t1**	K18703	SUGCT; succinate---hydroxymethylglutarate CoA-transferase [EC:2.8.3.13]	n	Mitochondrial
**g13694.t1**	K11498	CENPE; centromeric protein E	n	n
**g16639.t1**	K01875	SARS, serS; seryl-tRNA synthetase [EC:6.1.1.11]	n	Mitochondrial
**g12792.t1**	K08592	SENP1; sentrin-specific protease 1 [EC:3.4.22.68]	n	n
**g12837.t1**	K12821	PRPF40, PRP40; pre-mRNA-processing factor 40	n	n
**g13091.t1**	K24887	GTPBP1; GTP-binding protein 1	n	n
**g13095.t1**	K00939	adk, AK; adenylate kinase [EC:2.7.4.3]	n	n
**g13290.t1**	K06675	SMC4; structural maintenance of chromosome 4	n	n
**g13486.t1**	K08592	SENP1; sentrin-specific protease 1 [EC:3.4.22.68]	n	n
**g13520.t1**	K12821	PRPF40, PRP40; pre-mRNA-processing factor 40	n	n
**g13581.t1**	K15111	SLC25A26; solute carrier family 25 (mitochondrial S-adenosylmethionine transporter), member 26	n	Mitochondrial
**g13694.t1**	K11498	CENPE; centromeric protein E	n	n
**g13783.t1**	K12812	DDX39B, UAP56, SUB2; ATP-dependent RNA helicase UAP56/SUB2 [EC:3.6.4.13]	n	n
**g13784.t1**	K12812	DDX39B, UAP56, SUB2; ATP-dependent RNA helicase UAP56/SUB2 [EC:3.6.4.13]	n	n
**g13943.t1**	K01726	GAMMACA; gamma-carbonic anhydrase [EC:4.2.1.-]	n	Mitochondrial
**g13948.t1**	K08288	PRKCSH; protein kinase C substrate 80K-H	n	n
**g14169.t1**	K11090	LA, SSB; lupus La protein	n	n
**g14773.t1**	K11498	CENPE; centromeric protein E	n	n
**g15451.t1**	K00942	gmk, GUK1; guanylate kinase [EC:2.7.4.8]	n	n
**g15645.t1**	K06002	PGA; pepsin A [EC:3.4.23.1]	n	n
**g16100.t1**	K08288	PRKCSH; protein kinase C substrate 80K-H	n	n

**g16227.t1**	K08592	SENP1; sentrin-specific protease 1 [EC:3.4.22.68]	n	n
**g16320.t1**	K18932	ZDHHC; palmitoyltransferase [EC:2.3.1.225]	n	n
**g16412.t1**	K03469	rnhA, RNASEH1; ribonuclease HI [EC:3.1.26.4]	n	n
**g16639.t1**	K01875	SARS, serS; seryl-tRNA synthetase [EC:6.1.1.11]	Mitochondrial	n
**g17092.t1**	K15918	GLYK; D-glycerate 3-kinase [EC:2.7.1.31]	n	Chloroplast, mitochondria, nucleus
**g17171.t1**	K15111	SLC25A26; solute carrier family 25 (mitochondrial S-adenosylmethionine transporter), member 26	n	n
**g17341.t1**	K08592	SENP1; sentrin-specific protease 1 [EC:3.4.22.68]	n	n
**g17981.t1**	K05906	PCYOX1, FCLY; prenylcysteine oxidase / farnesylcysteine lyase [EC:1.8.3.5 1.8.3.6]	n	n
**g18240.t1**	K11498	CENPE; centromeric protein E	n	n
**g18296.t1**	K18932	ZDHHC; palmitoyltransferase [EC:2.3.1.225]	n	n
**g18501.t1**	K18932	ZDHHC; palmitoyltransferase [EC:2.3.1.225]	n	n
**g17341.t1**	K08592	SENP1; sentrin-specific protease 1 [EC:3.4.22.68]	n	n
**g18514.t1**	K08592	SENP1; sentrin-specific protease 1 [EC:3.4.22.68]	n	n
**g18609.t1**	K08592	SENP1; sentrin-specific protease 1 [EC:3.4.22.68]	n	n
**g19413.t1**	K07305	msrB; peptide-methionine (R)-S-oxide reductase [EC:1.8.4.12]	Mitochondrial	n
**g19479.t1**	K01726	GAMMACA; gamma-carbonic anhydrase [EC:4.2.1.-]	n	n
**g19493.t1**	K08288	PRKCSH; protein kinase C substrate 80K-H	n	n

Table S6. Presence/absence of vitamin C biosynthesis enzymes L-galactonolactone dehydrogenase (GLDH) and L-gulonolactone oxidase (GULO) in the genomes of six sequenced stramenopiles. Protein identifiers are also given for each hit.

**Species**	**GLDH**	**GULO**
Incisomonas marina	g8348.t1	g11511.t1
Schizochytrium aggregatum	98185	-
Phytophthora sojae	485337	-
Phaeodactylum tricornutum	23292	-
Blastocystis hominis Singapore Isolate B	-	-
Cafeteria roenbergensis BVI	622	7535

Table S7. Description of hits retrieved from the *Tara Oceans* Eukaryotic metagenome and single-cell assembled genome database (EUK_SMAGs) when using the predicted protein sequence for *I. marina* DSYB as a BLAST query, with an e-value cut-off of 1e^{-70}. The taxonomic classification of each MAG, metabolic mode and BUSCO completion as assigned by [16] is also given, with MAST taxa shaded in grey. Predicted subcellular targeting by HECTAR [15] is also given.

Protein hit Id.	MAG Id.	MAG Taxonomic Assignment by Delmont et al., 2022	Metabolic mode	BUSCO Completion (%)	Predicted targeting
SMAGs_714947	TARA_AON_82_MAG_00276_000000004952.4.1	MAST-7	Heterotrophic	30.2	Mitochondrial
SMAGs_9697997	TARA_ARC_108_MAG_00241_000000000996.1.1	MAST unclassified	Heterotrophic	38.8	Mitochondrial
SMAGs_2685109	TARA_ARC_108_MAG_00241_000000000996.1.1	MAST unclassified	Heterotrophic	38.8	Other
SMAGs_5412876	TARA_MED_95_MAG_00408_000000009893.2.1	New Oomycota	Heterotrophic	53.8	Other
SMAGs_5409111	TARA_MED_95_MAG_00408_000000009893.2.1	New Oomycota	Heterotrophic	53.8	Other
SMAGs_4706793	TARA_IOS_50_MAG_00124_000000001544.5.1	Aureococcus	Photosynthetic	54.9	Other
SMAGs_1750378	TARA_AOS_82_MAG_00144_000000003998.3.1	New_Chrysochromulinaceae_01	Photosynthetic	27.1	Mitochondrial
SMAGs_4614449	TARA_ION_45_MAG_00201_000000002677.5.1	New_Chrysochromulinaceae_01	Photosynthetic	74.9	Mitochondrial
SMAGs_6824365	TARA_PON_109_MAG_00232_000000000346.1.1	New_Chrysochromulinaceae_01	Photosynthetic	52.2	Mitochondrial
SMAGs_8443951	TARA_PSW_86_MAG_00280_000000000285.16.1	New_Chrysochromulinaceae_01	Photosynthetic	32.2	Mitochondrial
SMAGs_4027617	TARA_ARC_108_MAG_00326_000000006951.13.4	New_Chrysochromulinaceae_01	Photosynthetic	68.6	Mitochondrial
SMAGs_8506517	TARA_PSW_86_MAG_00285_000000000349.2.1	New_Chrysochromulinaceae_01	Photosynthetic	31.8	Signal peptide
SMAGs_720636	TARA_AON_82_MAG_00278_000000002804.3.1	New_Chrysochromulinaceae_01	Photosynthetic	27.8	Signal peptide
SMAGs_3784438	TARA_ARC_108_MAG_00319_000000002099.3.1	Phaeocystis	Photosynthetic	56.9	Mitochondrial
SMAGs_10058803	TARA_SOC_28_MAG_00074_000000006525.6.2	Phaeocystis	Photosynthetic	52.6	Mitochondrial
SMAGs_4145110	TARA_ION_45_MAG_00152_000000003198.10.1	Sister_Phaeocystis	Photosynthetic	43.6	Signal peptide
SMAGs_5732555	TARA_MED_95_MAG_00437_000000002734.2.2	Picozoa	Heterotrophic	21.6	Mitochondrial
SMAGs_3725435	TARA_ARC_108_MAG_00317_000000000411.3.1	Picozoa	Heterotrophic	75.7	Other
SMAGs_10171271	TARA_SOC_28_MAG_00079_000000015284.10.1	Picozoa	Heterotrophic	75.7	Mitochondrial
SMAGs_8949003	TARA_PSW_86_MAG_00314_000000005600.2.1	Picozoa	Heterotrophic	62.0	Mitochondrial
SMAGs_10199646	TARA_SOC_28_MAG_00079_000000002210.2.1	Picozoa	Heterotrophic	75.7	Other
SMAGs_2909318	TARA_ARC_108_MAG_00257_000000000227.17.2	Chonoflagellatea_NA	Heterotrophic	25.1	Mitochondrial
SMAGs_2830157	TARA_ARC_108_MAG_00250_000000008414.1.2	Chonoflagellatea_NA	Heterotrophic	40.4	Mitochondrial

SMAGs_5447382	TARA_MED_95_MAG_00409_000000011530.1.1	Ochrophyta_Dictyochophyceae_NA	Photosynthetic	72.6	Other

Table S8. Hits obtained from the *I. marina* MAG via BLAST using as query sequences genes for the four enzymes of DMSP degradation from *Roseobacter* spp. [17]. An e-value cut-off of 1e^{-70} was used. The numbers indicate how many orthologues were obtained, with 0 representing no hits.

	**KO**	K17486	K20034	K20035	K20036
	**KO_definition**	Dimethylsulfoniopro-pionate demethylase [EC:2.1.1.269]	3-(methylthio)-propionyl---CoA ligase [EC:6.2.1.44]	3-(methylthio)propanoyl-CoA dehydrogenase [EC:1.3.8.-]	(methylthio)acryloyl-CoA hydratase [EC:4.2.1.155]
	**gene**	*dmdA*	*dmdB*	*dmdC*	*dmdD/acuH*
**Bin**	**Taxonomy**				
1	*Incimonas marina*	0	0	0	1
2	*Pseudoalteromonas*	0	0	0	0
3	*Alteromonas sp.*	0	0	0	0
4	*Methylophaga sp.*	0	0	0	0
5	Unknown				
6	*Winogradskyella sediminis*	0	0	0	0
7	*Crocinitomicaceae*	0	0	0	0
8	*Arenibacter sp.*	0	0	0	0
9	*Muricauda sp.*	0	0	0	0
10	*Zhongshania sp002915595*	0	0	0	0
11	*Roseovarius halotolerans*	0	0	0	0
12	*Sulfitobacter marinus*	1	1	0	1
13	*Marinobacter hydrocarbonoclaticus*	0	0	0	0
14	*Alcanivorax sp.*	0	3	1	0
15	*Epibacterium scottomollicae*	1	0	0	0
16	*Antarctobacter heliothermus*	1	0	0	1
17	*Pararhizobium sp.*	0	0	0	0

18	*Hoeflea sp.*	0	1	0	0
19	*Hyphomonas atlantica*	0	0	0	0
20	*Thalassospira sp.*	0	0	1	0
21	*Idiomarina loihensis*	0	0	0	0
22	*Marinomonas sp.*	0	0	0	1
23	*Pseudooceanicola marinus*	0	0	0	0
24	*Parvibaculaceae**	0	1	1	0

Table S9. Accession numbers for the assembled metagenome sequences deposited in the European Nucleotide Archive (ENA)

Bin no.	Taxonomy	ENA Accession
1	*Incisomonas marina*	ERZ27257350
2	*Pseudoalteromonas*	ERZ27257351
3	*Alteromonas sp.*	ERZ27257354
4	*Methylophaga sp.*	ERZ27257356
5	*unknown*	ERZ27257360
6	*Winogradskyella seminidis*	ERZ27257566
7	*Crocinitomicaceae*	ERZ27257568
8	*Arenibacter sp.*	ERZ27257570
9	*Muricauda sp.*	ERZ27257571
10	*Zhongshania sp002915595*	ERZ27257573
11	*Roseovarius halotolerans*	ERZ27257575
12	*Sulfitobacter marinus*	ERZ27257577
13	*Marinobacter hydrocarbonoclaticus*	ERZ27257578
14	*Alcanivorax sp.*	ERZ27257580
15	*Epibacterium scottomollicae*	ERZ27257582
16	*Antarctobacter heliothermus*	ERZ27257583
17	*Pararhizobium sp.*	ERZ27257585
18	*Hoeflea sp.*	ERZ27257589
19	*Hyphomonas atlantica*	ERZ27257591
20	*Thalassospira sp.*	ERZ27257593
21	*Idiomarina loihensis*	ERZ27257594
22	*Marinomonas sp.*	ERZ27257595
23	*Pseudooceanicola marinus*	ERZ27257596
24	*Parvibaculaceae*	ERZ27257597
Whole meta-genome	Not applicable	ERZ27257279

Supplementary References

1. **Ranallo-Benavidez TR, Jaron KS, Schatz MC**. GenomeScope 2.0 and Smudgeplot for reference-free profiling of polyploid genomes. *Nature Communications 2020 11:1* 2020;11:1–10.
2. **Thakur R, Shiratori T, Ishida K**. Taxon-rich Multigene Phylogenetic Analyses Resolve the Phylogenetic Relationship Among Deep-branching Stramenopiles. *Protist* 2019;170:125682.
3. **Hackl T, Martin R, Barenhoff K, Duponchel S, Heider D, *et al.*** Four high-quality draft genome assemblies of the marine heterotrophic nanoflagellate Cafeteria roenbergensis. *Scientific Data 2020 7:1* 2020;7:1–9.
4. **Denoeud F, Roussel M, Noel B, Wawrzyniak I, Da Silva C, *et al.*** Genome sequence of the stramenopile Blastocystis, a human anaerobic parasite. *Genome Biol*;12. Epub ahead of print 25 March 2011. DOI: 10.1186/GB-2011-12-3-R29.
5. **Bowler C, Allen AE, Badger JH, Grimwood J, Jabbari K, *et al.*** The Phaeodactylum genome reveals the evolutionary history of diatom genomes. *Nature* 2008;456:239–244.
6. **Tyler BM, Tripathy S, Zhang X, Dehal P, Jiang RHY, *et al.*** Phytophthora genome sequences uncover evolutionary origins and mechanisms of pathogenesis. *Science (1979)* 2006;313:1261–1266.
7. **Liang L, Zheng X, Fan W, Chen D, Huang Z, *et al.*** Genome and Transcriptome Analyses Provide Insight Into the Omega-3 Long-Chain Polyunsaturated Fatty Acids Biosynthesis of Schizochytrium limacinum SR21. *Front Microbiol* 2020;11:519814.
8. **Wilkens SL, Maas EW**. Development of a novel technique for axenic isolation and culture of thraustochytrids from New Zealand marine environments. *J Appl Microbiol* 2012;112:346–352.
9. **Droop MR**. A procedure for routine purification of algal cultures with antibiotics. *British Phycological Bulletin* 1967;3:295–297.
10. **Ferrante M, Annunziata R, Russo MT, Manfellotto F**. Axenic Diatoms cultures protocol. *Protocols.io*. https://dx.doi.org/10.17504/protocols.io.bgudjws6 (2020, accessed 28 July 2021).
11. **Koedooder C, Stock W, Willems A, Mangelinckx S, De Troch M, *et al.*** Diatom-bacteria interactions modulate the composition and productivity of benthic diatom biofilms. *Front Microbiol*;10. Epub ahead of print 2019. DOI: 10.3389/FMICB.2019.01255/FULL.
12. **Derelle R, López-García P, Timpano H, Moreira D**. A Phylogenomic Framework to Study the Diversity and Evolution of Stramenopiles (=Heterokonts). *Mol Biol Evol* 2016;33:2890–2898.
13. **Eren AM, Esen OC, Quince C, Vineis JH, Morrison HG, *et al.*** Anvi'o: an advanced analysis and visualization platform for 'omics data. *PeerJ*;3. Epub ahead of print 2015. DOI: 10.7717/PEERJ.1319.

14. **Kanehisa M, Sato Y, Morishima K, Sternberg M**. BlastKOALA and GhostKOALA: KEGG Tools for Functional Characterization of Genome and Metagenome Sequences. *J Mol Biol*;428. Epub ahead of print 2015. DOI: 10.1016/j.jmb.2015.11.006.

15. **Gschloessl B, Guermeur Y, Cock JM**. HECTAR: A method to predict subcellular targeting in heterokonts. *BMC Bioinformatics* 2008;9:1–13.

16. **Delmont TO, Gaia M, Hinsinger DD, Frémont P, Vanni C, *et al.*** Functional repertoire convergence of distantly related eukaryotic plankton lineages abundant in the sunlit ocean. *Cell genomics*;2. Epub ahead of print 11 May 2022. DOI: 10.1016/J.XGEN.2022.100123.

17. **Shao X, Cao HY, Zhao F, Peng M, Wang P, *et al.*** Mechanistic insight into 3-methylmercaptopropionate metabolism and kinetical regulation of demethylation pathway in marine dimethylsulfoniopropionate-catabolizing bacteria. *Mol Microbiol* 2019;111:1057–1073.
